# Pd-Catalyzed Formal [2 + 2]-Retrocyclization of Cyclobutanols via 2-Fold Csp^3^–Csp^3^ Bond Cleavage

**DOI:** 10.1021/acs.joc.3c01750

**Published:** 2024-01-04

**Authors:** Sergio Parra-García, Marina Ballester-Ibáñez, José-Antonio García-López

**Affiliations:** Grupo de Química Organometálica, Departamento de Química Inorgánica, Facultad de Química, Universidad de Murcia, E-30100 Murcia, Spain

## Abstract

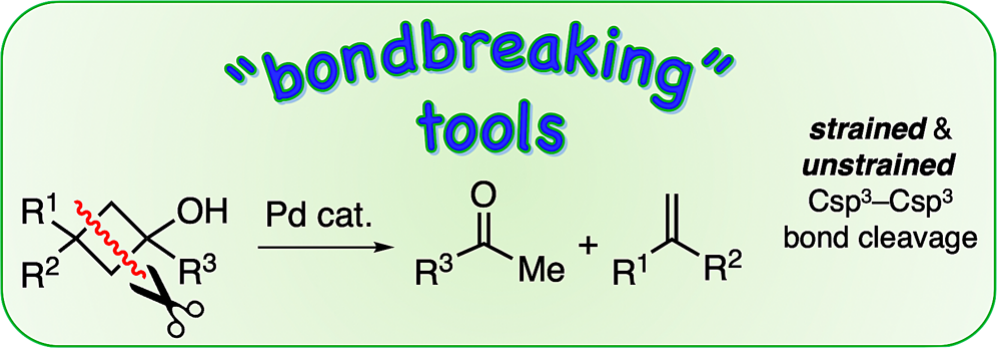

In this work, we describe the unexpected 2-fold Csp^3^–Csp^3^ bond cleavage suffered by cyclobutanols in the presence of a catalytic amount of Pd(OAc)_2_ and promoted by the bulky biaryl JohnPhos ligand. Overall, the sequential cleavage of a strained and an unstrained Csp^3^–Csp^3^ bond leads to the formal [2 + 2]-retrocyclization products, namely, styrene and acetophenone derivatives. This procedure might enable the use of cyclobutanols as masked acetyl groups, resisting harsh conditions in organic synthesis.

## Introduction

**Scheme 1:**
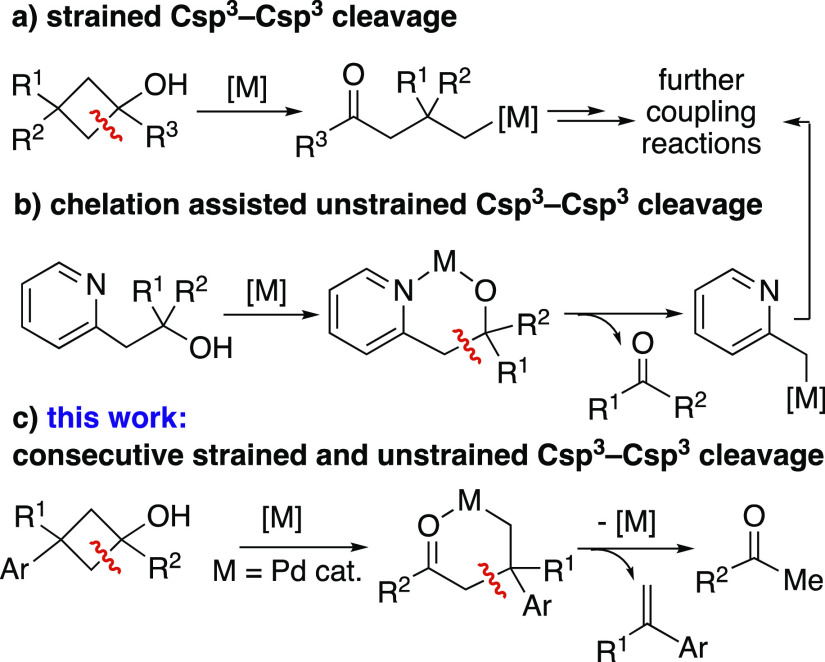
Literature Precedents and Novel Work on Csp^3^–Csp^3^ Bond Cleavage

The study and development of reactions relying on the cleavage of C–C bonds has attracted great attention over the last years.^1^ The deeper understanding of these processes has allowed the design of new routes in organic synthesis as well as the diversification in the applications of certain building blocks.^2−5^ One of the main avenues of research in this field has focused on the use of strained starting materials such as cyclobutanols.^6−8^ It is well-established that these scaffolds are suitable substrates for transition-metal catalysis, given their tendency to undergo β-C elimination upon coordination of the alcohol moiety to metals such as Rh, Pd, or Ni.^8−11^ Hence, the opening of the strained cyclobutyl ring gives rise to a sigma-alkyl organometallic intermediate, which can suffer different transformations depending on the type of the metallic catalyst, the substrate substitution pattern, and the specific reaction conditions (Scheme 1, a). These transformations may include β-H elimination, ring expansion or ring contraction processes, among others.^12^

The higher bond enthalpy of unstrained Csp^3^–Csp^3^ linkages compared to those present in strained molecules renders the cleavage of the first ones a challenging goal.^13−16^ One of the approaches to achieve β-alkyl elimination in unstrained substrates through TM-catalysis relies on the introduction of auxiliary coordinating or chelating groups within the molecular skeleton of the substrate. Thus, a range of alcohols bearing a coordinating moiety conveniently located in its structure (pyridyl,^17,18^ pyridyl-*N*-oxide,^19^ ketone,^20,21^ azide,^22^ or allyl^23^ groups) have been successfully derivatized through Csp^3^–Csp^3^ cleavage (Scheme 1, b). Other chelating groups like 8-aminoquinoline have also been reported to assist β-alkyl elimination in Pd-mediated or catalyzed systems.^24,25^

In every TM-catalyzed process, the activity of a catalyst and the proper chemoselectivity of the transformation can be extraordinarily affected by the ancillary ligands, which may enable the tuning of a reaction’s outcome. This is the case of widely used phosphine ligands, which are commercially available scaffolds exhibiting significant variation on their electronic and steric properties.^26,27^ For instance, bulky biaryl-monophosphine ligands are able to promote unique reactivity patterns, especially in Pd-catalyzed cross-coupling reactions, assisting the activation or the formation of C–heteroatom bonds.^28−31^ During the course of our previous studies on processes involving Pd-catalyzed C–C bond cleavage of cyclobutanols and their application to organic synthesis,^32^ we observed that the use of a bulky phosphine ligand such as (2-biphenyl)di-*tert*-butylphosphine, known as JohnPhos, not only failed to deliver the expected coupling products in good yields but also produced the degradation of the starting cyclobutanol reagent. Intrigued by this observation, we decided to further investigate this curious behavior involving a 2-fold Csp^3^–Csp^3^ bond cleavage (Scheme 1, c), the main features of which are discussed in the present manuscript.

## Results and Discussion

**Scheme 2:**
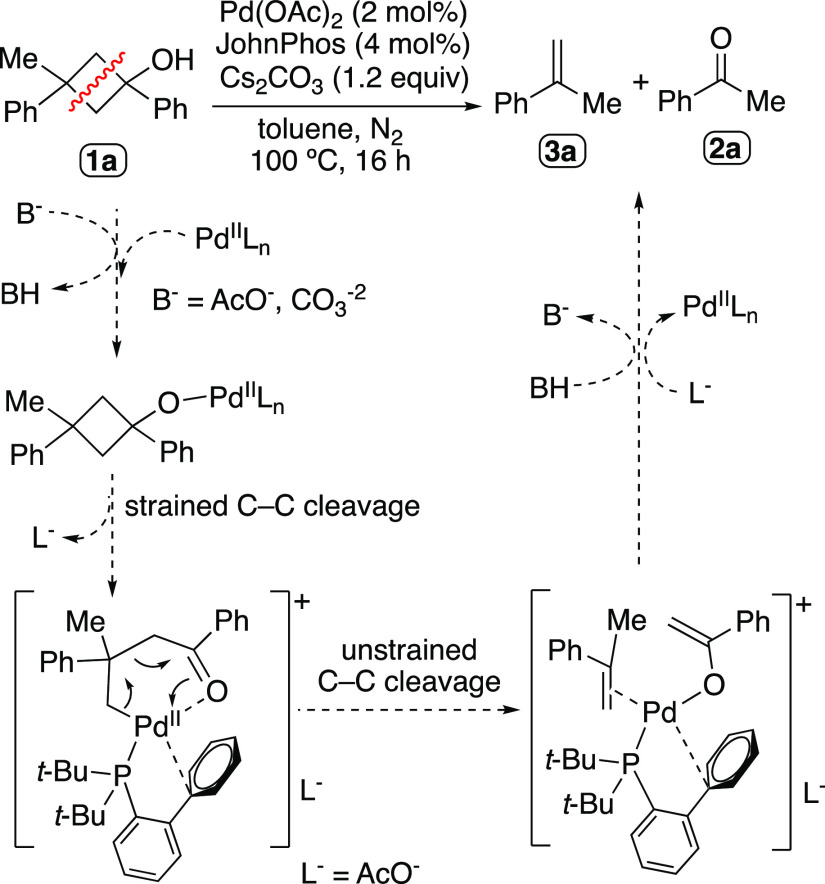
Observed Reactivity of Cyclobutanol Derivatives

In order to study the reactivity of tertiary cyclobutanol derivatives toward the Pd(II)/Johnphos catalytic system, we performed an initial experiment by heating a mixture of both diastereoisomers of the cyclobutanol substrate **1a** in toluene at 100 °C in the presence of 2 mol % of Pd(OAc)_2_, 4 mol % of JohnPhos, and Cs_2_CO_3_ (1.2 equiv) and in the absence of any other additional coupling reagents. To our delight, the behavior previously observed in other cross-coupling reaction mixtures involving cyclobutanol derivatives as alkylating reagents was reproducible; that is, the strained alcohol had been completely consumed in a clean process, leading to a mixture of two main components that were easily identified by ^1^H NMR spectroscopy as the acetophenone **2a** and the α-methylstyrene **3a** (Scheme 2).

The outcome of this catalytic transformation implies a two-fold Csp^3^–Csp^3^ bond cleavage within the cyclobutanol scaffold. While there are many synthetic protocols that exploit the cleavage of a certain C–C bond within the molecular skeleton, the processes in which two or more of these linkages are cleaved in a single reaction are rather limited,^33−37^ especially if they involve Csp^3^–Csp^3^ bonds.^38−44^ Some examples are the thermal [2 + 2]-retrocyclization reactions taking place on cyclobutane derivatives, processes that normally occur in strained structures embedding a saturated four-membered ring.^38−43,45^ In our case, the first Csp^3^–Csp^3^ bond cleavage would respond to the expected and well-known opening of the strained carbocycle through a β-C elimination pathway (Scheme 2),^6,46^ while the second Csp^3^–Csp^3^ bond splitting event would take place on an unstrained sigma-alkyl intermediate. In this case, either a β-C elimination or a plausible retrocyclization mechanism assisted by the ketone group could operate to render α-methylstyrene **3a** along with a Pd(II) enolate, which would finally undergo a protodepalladation step to afford acetophenone **2a** and restore the catalytic Pd(II) species. Goeke et al. reported a related [2 + 2] cycloreversion process occurring in alkylation reactions of fused cyclobutanones with organolithium reagents.^47^ The possible assistance of the ketone group to facilitate the Csp^3^–Csp^3^ bond cleavage event would be analogous to that one formerly described in Pd-catalyzed retro-aldol reactions;^20^ nevertheless, in those cases a six-membered *O*,*O*-palladacycle intermediate is formed, instead of a *C*,*O*-palladacycle as might happen in our case.

We tested that the 2-fold C–C cleavage process was not proceeding in the absence of either Pd(OAc)_2_, Cs_2_CO_3_, or JohnPhos, with the recovery of unreacted starting material. Similarly, no reaction took place when Et_3_N was used as the base, or when JohnPhos was replaced by PPh_3_. The use of a bulky phosphine ligand with a related 2-biaryl motif such as 2-dicyclohexylphosphino-2′,6′-dimethoxybiphenyl, known as SPhos, provided the expected C–C cleavage products albeit in lower yield compared to the reactions performed with JohnPhos. Therefore, this transformation seems to be promoted by the particular coordination environment of a Pd center bearing a bulky biarylphosphine, in particular, the JohnPhos ligand. We believe that the bulkiness of this phosphine, along with its ability to coordinate through π-interaction of the biaryl motif, may force the decoordination of an acetate ligand and promote the formation of the *C*,*O*-chelate intermediate from which the enolate formation occurs (Scheme 2).

**Scheme 3:**
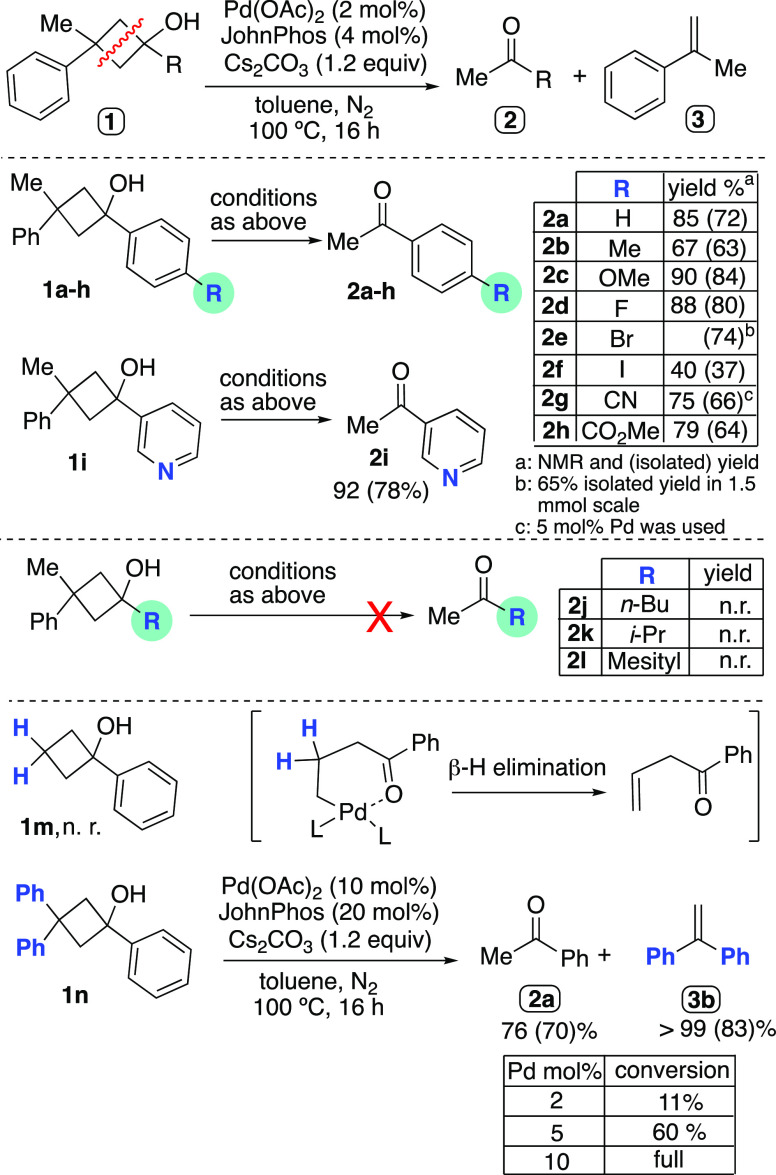
2-Fold Csp^3^–Csp^3^ Cleavage in Cyclobutanols with Different Substitution Patterns

Once that the experimental conditions leading to full conversion of the starting cyclobutanol into its scission products **2** and **3** were established, we studied this formal [2 + 2]-retrocycloaddition reaction employing a range of tertiary cyclobutanols with different substitution patterns (Scheme 3). Essentially, the reaction tolerated well the presence of electron-donating [Me (**1b**), OMe (**1c**)] and electron-withdrawing [F (**1d**), Br (**1e**), I (**1f**); CN (**1g**), and CO_2_Me (**1h**)] groups on the aryl ring, leading in all cases to clean conversion (≥99%) of the substrates into the acetophenones **2b–h** and the styrene **3a**. The ^1^H NMR yields of **2b–h** were slightly lower due to their partial loss in the workup process, given the relative volatility of these compounds, a fact that is also resembled in the smaller amounts detected for **3a** in the crude mixtures (e.g., ^1^H NMR yields of **2a** and **3a** were 85 and 46%, respectively). The cyano derivative **1g** required a slightly higher Pd loading (5 mol %) to reach full conversion, likely due to competing coordination of the CN group to Pd. The bromine atom of substrate **1e** remained intact under these conditions; however, the more reactive iodinated derivative **1f** afforded only a moderated yield, probably due to further coupling reactions caused by Pd(0) species generated upon ultimate degradation of the catalyst. The pyridyl derivative **1i** was also productive, yielding 92% of the desired 4-acetylpyridine. In contrast, those substrates bearing an alkyl group [*n*-Bu (**1j**) and *i*-Pr (**1k**)] on the hydroxylated carbon of the cyclobutyl ring failed to deliver the expected products, being recovered unreacted. When forcing the conditions by using 5 mol % of Pd loading and heating to 130 °C, a modest 20% conversion of **1j** was observed. No reaction took place when sterically hindered mesityl derivative **1l** was used as the substrate.

The viability of cyclobutanols with different substitution patterns on the strained carbocycle was also assessed for this transformation. When the substrate **1m** was used as starting material, no reaction was observed. Probably, the corresponding σ-alkyl Pd(II) intermediate might undergo β-H elimination, leading to inactive Pd(0) species (Scheme 3). The triarylated cyclobutanol **1n** required up to 10 mol % Pd(OAc)_2_ load to be completely transformed into the corresponding 1,1-diphenylethylene and acetophenone, perhaps due to the higher steric hindrance of the substituents, which might hamper the adoption of the adequate conformation of the key organometallic intermediate.

**Scheme 4:**
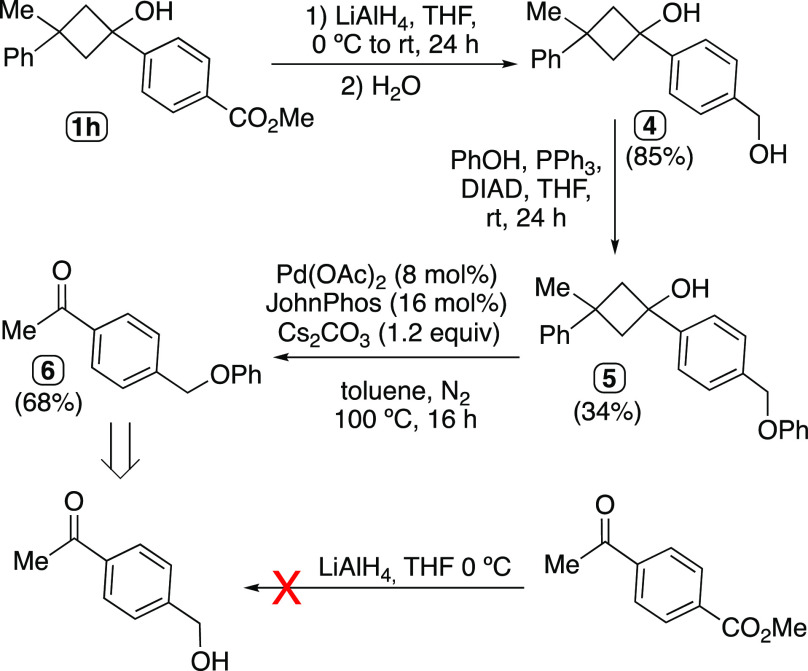
Cyclobutanol as a Masked Acetyl Group Resisting the Attack of Strong Reducing Agents

Although mechanistically interesting, the 2-fold Csp^3^–Csp^3^ bond cleavage of cyclobutanol cannot be considered as a proper synthetic route to get neither α-methylstyrenes nor acetophenones, commercially available compounds that can be prepared by much simpler and economical routes. Nevertheless, we envisioned that this catalytic reaction could find applications in organic chemistry as a complement to protecting groups in synthetic strategies. For instance, the cyclobutanol moiety could be considered a masked acetyl group. As a representative example, we utilized the starting material **1h**, bearing an ester moiety, in order to carry out several transformations on the carboxymethyl moiety under conditions that an acetyl group could not tolerate. When **1h** was stirred in THF in the presence of a strong reducing agent such as LiAlH_4_, the ester group was easily converted into the corresponding primary alcohol to render the diol **4** (Scheme 4), which could undergo a further transformation, for instance through a Mitsunobu reaction to give the derivative **5**. The submission of **5** to the catalytic conditions for the 2-fold C–C cleavage rendered the desired acylated compound **6** in good yield after the workup. An alternative route to reach **6** could involve the use of 4-acetylbenzyl alcohol; however, this starting material could not be obtained selectively by direct reduction of the unprotected methyl 4-acetylbenzoate precursor material with LiAlH_4_.

**Scheme 5:**
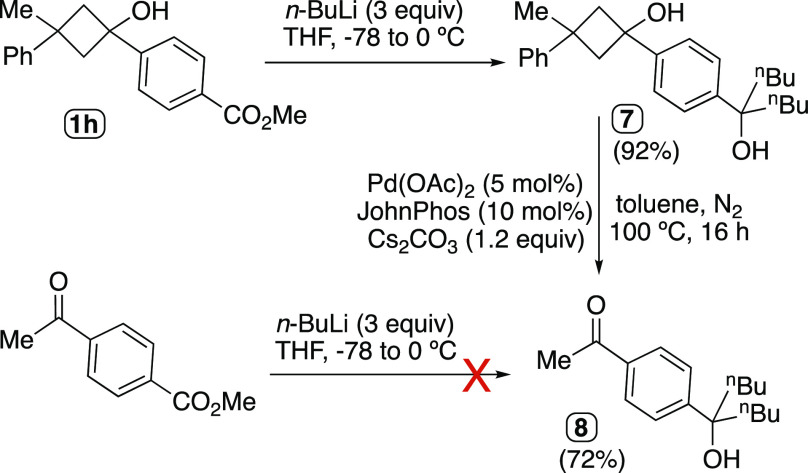
Cyclobutanol as a Masked Acetyl Group Resisting the Attack of Strong Nucleophiles

One of the classic transformations in organic chemistry involving carbonyl group reactivity relies on the addition of nucleophiles such as organometallic reagents. The diol **7** could be easily obtained by reaction of the cyclobutanol derivative **1h** with excess of *n*-BuLi (Scheme 5). The cyclobutanol moiety present in **7** could then be smoothly transformed into the acetyl group under the Pd(OAc)_2_/JohnPhos catalytic conditions. As happened in the case of compound **6**, the derivative **8** would not be obtained chemoselectively from the reaction of unprotected methyl 4-acetylbenzoate with an excess of *n*-BuLi, given the well-known electrophilic character of the ketone moiety.

In summary, we describe here the ability of a Pd/JohnPhos catalytic system to cleave two Csp^3^–Csp^3^ bonds of substituted cyclobutanol substrates, involving the sequential strained and unstrained cleavage of such bonds. Furthermore, the in situ generated ketone moiety might assist the splitting of the unstrained C–C bond mimicking the mechanism proposed for Pd-catalyzed retroaldol reactions. In addition, these results point to the consideration of the cyclobutanol moiety as a masked acetyl group resisting harsh reaction conditions.

## Data Availability

The data underlying this study are available in the published article and its Supporting Information.

## Supporting Information Available

Experimental procedures, characterization data, and NMR spectra of the new compounds (PDF)
