# Supplementary material for: Pd-Catalyzed Formal [2 + 2]-Retrocyclization of Cyclobutanols via 2-Fold Csp3–Csp3 Bond Cleavage
Source: J Org Chem. 2024 Jan 4;89(2):882–6. doi: 10.1021/acs.joc.3c01750 (PMC10804411; doi:10.1021/acs.joc.3c01750)
Supplement: Supplementary file 1 — jo3c01750_si_001.pdf [file jo3c01750_si_001.pdf]

SUPPORTING INFORMATION for

Pd-catalyzed formal [2+2]-retrocyclization of cyclobutanols via two-fold Csp^3–Csp^3 bond cleavage

Sergio Parra-García,[1] Marina Ballester-Ibáñez,[1] and José-Antonio García-López*,[1]

[1]Grupo de Química Organometálica, Departamento de Química Inorgánica, Facultad de Química, Universidad de Murcia, E-30100 Murcia, Spain.

E-mail: joangalo@um.es

Synthesis of cyclobutanol starting materials S1

Characterization data of the starting materials S4

Representative procedure and optimization table for two-fold C-C cleavage reaction S10

Scope of the two-fold C-C cleavage reaction S11

References S13

NMR spectra of the non-previously reported compounds S14

NMR spectra of crude reaction mixture arising from the two-fold C-C bond cleavage procedure and purified products S26

General Remarks

Infrared spectra were recorded on a Perkin-Elmer spectrum 100 spectrophotometer. High-resolution ESI mass spectra were recorded on an Agilent 6220 Accurate Mass TOF LC/MS spectrometer. Nuclear Magnetic Resonance (NMR) spectra were recorded on a 300 or 400 MHz Bruker NMR spectrometers in $CDCl_3$ at 298 K (unless stated otherwise). All chemical shift values are reported in parts per million (ppm) with coupling constant (*J*) values reported in Hz. All spectra were referenced to TMS for ^1H NMR and the $CDCl_3$ solvent peak for $^{13}C\{^1H\}$ NMR. Structural assignments were made with additional information from ^{13}C-APT experiments. Anhydrous solvents were purchased from commercial sources and used as received. TLC tests were run on TLC Alugram® Sil G plates and visualized under UV light at 254 nm. Chromatography: Separations were carried out on silica gel.

Synthesis of cyclobutanol starting materials

Representative procedure A

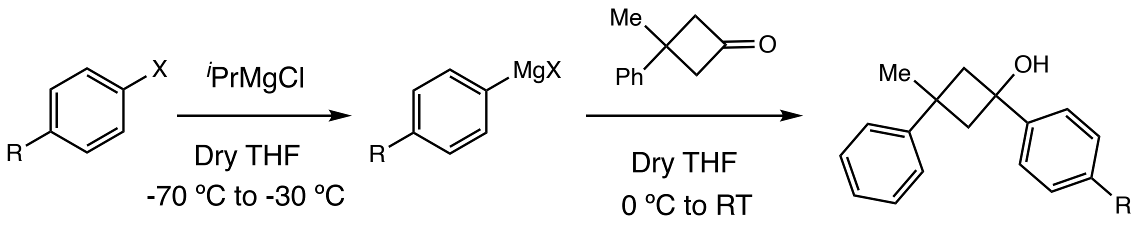

A solution of commercially available isopropylmagnesium chloride in dry THF (1.1 equiv.) was added dropwise under N_2 atmosphere to a solution of the corresponding aryl halide (1.05 equiv.) in dry THF at −70 °C. After 20 minutes of stirring, the bath temperature was increased to −30 °C and the mixture was further stirred for 20 minutes. The resulting solution was added dropwise to a solution of 3-methyl-3-phenylcyclobutan-1-one (1 equiv.) in dry THF at 0 °C under N_2 atmosphere. The reaction was warmed to RT and stirred (see the characterization data of starting materials to check the reaction time). The reaction was quenched with water, the mixture was filtered, and the solid residue was washed with Et_2O (2 × 5 mL). The filtrated was concentrated to ca. 2 mL, diluted with Et_2O (50 mL) and washed with water (30 mL). The aqueous layer was extracted with Et_2O (2 × 20 mL) and the combined organic layers were washed with brine, dried over $MgSO_4$, filtered, and concentrated in vacuum. The product was precipitated as a white solid with *n*-pentane, filtered and dried under reduced pressure or purified by flash column chromatography with *n*-hexane and EtOAc as eluents.

Experimental procedure for the synthesis of cyclobutanol 1l

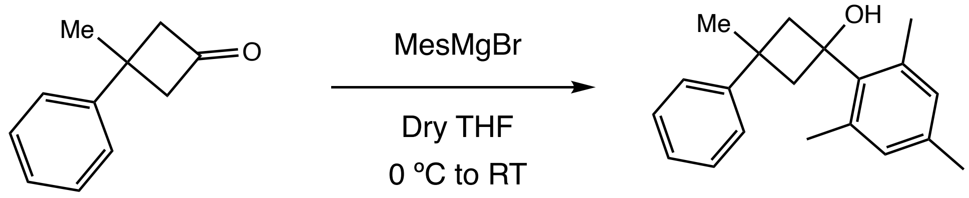

A solution of commercially available 2-mesitylmagnesium bromide in dry THF (8 mL, 5.20 mmol, 1.3 eq.) was added dropwise to a solution of 3-methyl-3-phenylcyclobutan-1-one (624 mg, 4.00 mmol, 1 equiv.) in dry THF at 0 °C under N_2 atmosphere. The reaction was warmed to RT and stirred for 6 hours. The reaction was quenched with water, the mixture was filtered, and the solid residue

was washed with Et_2O (2 × 5 mL). The filtrated was concentrated to ca. 2 mL, diluted with Et_2O (50 mL) and washed with water (30 mL). The aqueous layer was extracted with Et_2O (2 × 20 mL) and the combined organic layers were washed with brine, dried over $MgSO_4$, filtered, and concentrated in vacuum. The product was purified by flash column chromatography with *n*-hexane and EtOAc as eluents to give compound **1l** as a colourless oil (666 mg, 2.38 mmol, 59%).

Experimental procedure for the synthesis of cyclobutanol 4

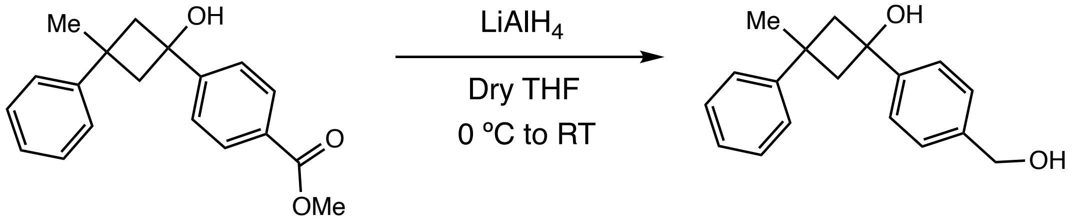

A solution of methyl 4-(1-hydroxy-3-methyl-3-phenylcyclobutyl)benzoate (**1h**, 296 mg, 1.00 mmol, 1 equiv.) in dry THF under N_2 atmosphere was added dropwise to a suspension of $LiAlH_4$ (169 mg, 5.00 mmol, 5 equiv.) in dry THF under N_2 atmosphere at 0 °C. The reaction was stirred at RT overnight and quenched slowly with water in an ice bath at 0 °C. The mixture was decanted, and the liquid residue was concentrated to ca. 2 mL, diluted with AcOEt (50 mL) and washed with water (50 mL). The aqueous layer was extracted with EtOAc (2 × 25 mL), and the combined organic layers were washed with brine, dried over $MgSO_4$, filtered, and concentrated in vacuum. The product was purified by flash column chromatography with *n*-hexane and EtOAc as eluents to give compound **4** as a white solid (228 mg, 0.85 mmol, 85%).

Experimental procedure for the synthesis of cyclobutanol 5

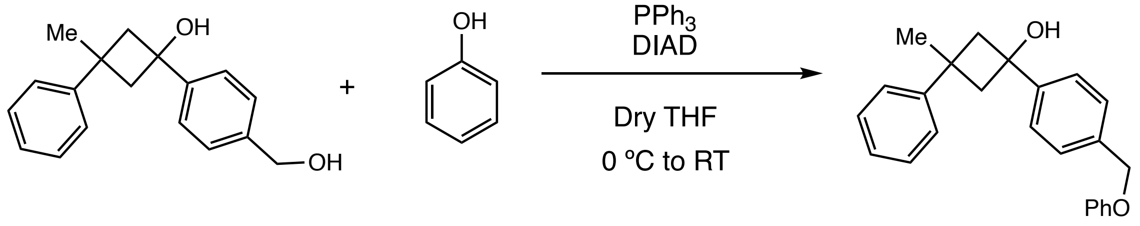

Diisopropylazodicarboxylate (DIAD, 376 μL, 1.9 mmol, 1 equiv.) was added to a solution of 1-(4-(hydroxymethyl)phenyl)-3-methyl-3-phenylcyclobutan-1-ol (**4**,

513 mg, 1.9 mmol, 1 equiv.), PPh_3 (501 mg, 1.9 mmol, 1 equiv.) and phenol (167 mg, 1.8 mmol, 0.95 equiv.) in dry THF at 0 °C. The reaction was stirred for 15 minutes at 0 °C, warmed to RT and stirred for 24 hours. The mixture was concentrated under reduced pressure and the residue was purified by flash column chromatography with *n*-hexane and EtOAc as eluents to give compound **5** as a white solid (211 mg, 0.61 mmol, 34%).

Experimental procedure for the synthesis of cyclobutanol 7

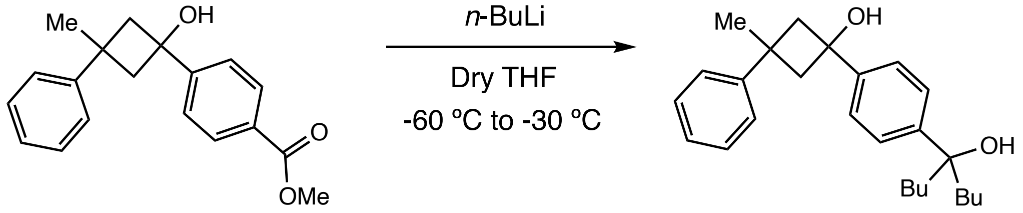

A solution of *n*-butyllithium in dry hexanes (3.6 mL, 5.76 mmol, 1 equiv.) was added dropwise under N_2 atmosphere to a solution of methyl 4-(1-hydroxy-3-methyl-3-phenylcyclobutyl)benzoate (**1h**, 505 mg, 1.7 mmol, 1 equiv.) in dry THF at −60 °C. After 10 minutes, 2.4 equiv. of *n*-butyllithium was added dropwise to the reaction and the mixture was stirred at −30 °C for 2 hours. The reaction was quenched with water and the mixture was concentrated under vacuum to ca. 2 mL, diluted with EtOAc (50 mL) and washed with water (30 mL). The aqueous layer was extracted with EtOAc (2 × 25 mL), and the combined organic layers were washed with brine, dried over $MgSO_4$, filtered, and concentrated in vacuum. The product was purified by flash column chromatography with *n*-hexane and EtOAc as eluents to give compound **7** as an off white waxy solid (597 mg, 1.57 mmol, 92%).

Characterization data of the starting materials

Compounds **1a**, **1c**, **1j**, **1k** and **1n** were obtained following the representative procedure **A** and they were previously described in the literature.[1] Similarly, compounds **1b** and **1m** were obtained following the representative procedure **A** and they were also previously described in the literature.[2, 3]

Compound 1-(4-fluorophenyl)-3-methyl-3-phenylcyclobutan-1-ol (1d) was

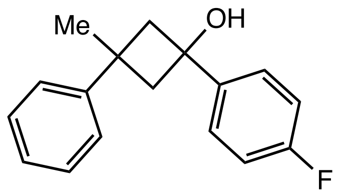

obtained from 1-fluoro-4-iodobenzene (466 mg, 2.10 mmol), isopropylmagnesium chloride (1.2 mL, 2.20 mmol) and 3-methyl-3-phenylcyclobutan-1-one (312 mg, 2.00 mmol) following the representative procedure **A** as a white solid by precipitation after 3 hours of reaction time (153 mg, 0.60 mmol, 30%). The product was isolated and characterized as a mixture of diastereoisomers in 1:0.84 ratio. IR (cm^{-1}): ν 3208, 2978, 2930, 1602, 1508, 1222, 1159, 830, 764, 701, 544. ^{1}H-NMR (300 MHz, $CDCl_3$): δ 7.54 − 7.48 (m, 1.9 H), 7.35 – 7.04 (m, 12.8 H), 6.98 – 6.92 (m, 1.7 H), 2.99 – 2.85 (m, 5.5 H), 2.63 – 2.59 (m, 1.7 H), 1.98 (s, 0.8 H), 1.87 (s, 1 H), 1.70 (s, 2.5 H), 1.26 (s, 3 H). ^{13}C{^{1}H}-NMR (75.45 MHz, $CDCl_3$): δ 162.1 (d, $^1J_{CF}$ = 249.0 Hz, C_q), 161.8 (d, $^1J_{CF}$ = 249.0 Hz, C_q), 151.4 (s, C_q), 143.2 (d, $^4J_{CF}$ = 3.0 Hz, C_q), 142.2 (d, $^4J_{CF}$ = 3.0 Hz, C_q), 128.4 (s, CH), 128.3 (s, CH), 127.6 (d, $^3J_{CF}$ = 7.5 Hz, CH), 126.6 (d, $^3J_{CF}$ = 7.5 Hz, CH), 125.6 (s, CH), 125.4 (s, CH), 125.2 (s, CH), 125.1 (s, CH), 115.4 (d, $^2J_{CF}$ = 22.6 Hz, CH), 115.1 (d, $^2J_{CF}$ = 22.6 Hz, CH), 72.5 (s, C_q), 72.1 (s, C_q), 49.1 (s, CH_2), 48.6 (s, CH_2), 35.9 (s, C_q), 34.3 (s, C_q), 32.7 (s, CH_3), 31.4 (s, CH_3). One C_q signal of both diastereoisomers is overlapped. ^{19}F{^{1}H}-NMR (282.4 MHz, $CDCl_3$): δ –115.1 (s), –115.8 (s). GC-MS *m/z* calculated for $C_{17}H_{17}FO$ $[M]^+$ 256.1263, found 256.1257.

Compound 1-(4-bromophenyl)-3-methyl-3-phenylcyclobutan-1-ol (1e) was

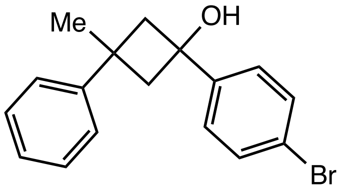

obtained from 1-bromo-4-iodobenzene (3.370 g, 11.90 mmol), isopropylmagnesium chloride (7.8 mL, 12.49 mmol) and 3-methyl-3-phenylcyclobutan-1-one (1.769 g, 11.34 mmol) following the representative procedure **A** as a white solid by precipitation after 3 hours of reaction time (2.220 g, 7.00 mmol, 62%). The product was obtained and characterized as a mixture of diastereoisomers in 1:1 ratio. IR (cm^{-1}): ν 3274, 2964, 1487, 1005, 830, 821, 765, 700, 546. ^{1}H-NMR (300 MHz, $CDCl_3$): δ 7.53 − 7.50 (m, 2 H), 7.41 – 7.27 (m, 10 H), 7.18 – 7.14 (m, 6 H), 2.94 – 2.81 (m, 6 H), 2.59 – 2.54 (m, 2 H), 2.15 (s, 1 H), 2.03 (s, 1 H), 1.68 (s, 3 H), 1.25 (s, 3 H). ^{13}C{^{1}H}-NMR (75.45 MHz, $CDCl_3$): δ 151.2 (s, C_q), 146.3 (s, C_q), 145.3 (s, C_q), 131.6 (s, CH), 131.3 (s, CH), 128.4 (s, CH), 128.2 (s, CH), 127.6 (s, CH), 126.6 (s, CH), 125.6 (s, CH), 125.4 (s, CH),

125.2 (s, CH), 125.0 (s, CH), 121.4 (s, C_q), 120.8 (s, C_q), 72.5 (s, C_q), 72.0 (s, C_q), 48.9 (s, CH_2), 48.5 (s, CH_2), 35.9 (s, C_q), 34.3 (s, C_q), 32.8 (s, CH_3), 31.5 (s, CH_3). One C_q signal of both diastereoisomers is overlapped. HR-MS (+ESI) *m/z* calculated for $C_{17}H_{16}Br$ $[M-OH]^+$ 299.0435, found 299.0430.

Compound 1-(4-iodophenyl)-3-methyl-3-phenylcyclobutan-1-ol (1f) was

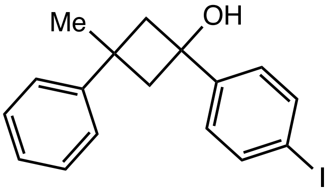

obtained from 1,4-diiodobencene (3.464 g, 10.50 mmol), isopropylmagnesium chloride (5.3 mL, 10.52 mmol) and 3-methyl-3-phenylcyclobutan-1-one (1.560 g, 10.00 mmol) following the representative procedure **A** as a white solid by precipitation after 3 hours of reaction time (1.969 g, 5.41 mmol, 54%). The product was obtained and characterized as a mixture of diastereoisomers in 1:1 ratio. IR (cm^{-1}): ν 3279, 2961, 1484, 1442, 1417, 1387, 1222, 1104, 1018, 1002, 893, 829, 814, 764, 697, 544, 477. ^{1}H-NMR (300 MHz, $CDCl_3$): δ 7.76 – 7.72 (m, 2 H), 7.62 – 7.57 (m, 2 H), 7.38 – 7.27 (m, 8 H), 7.23 – 7.14 (m, 4 H), 7.08 – 7.04 (m, 2 H), 2.96 – 2.88 (m, 6 H), 2.62 – 2.57 (m, 2 H), 1.98 (s, 1 H), 1.86 (s, 1 H), 1.70 (s, 3 H), 1.27 (s, 3 H). ^{13}C{^{1}H}-NMR (75.45 MHz, $CDCl_3$): δ 151.2 (s, C_q), 147.1 (s, C_q), 146.0 (s, C_q), 137.6 (s, CH), 137.4 (s, CH), 128.4 (s, CH), 128.3 (s, CH), 127.8 (s, CH), 126.9 (s, CH), 125.6 (s, CH), 125.5 (s, CH), 125.2 (s, CH), 125.0 (s, CH), 93.1 (s, C_q), 92.5 (s, C_q), 72.6 (s, C_q), 72.2 (s, C_q), 48.9 (s, CH_2), 48.6 (s, CH_2), 35.9 (s, C_q), 34.4 (s, C_q), 32.8 (s, CH_3), 31.5 (s, CH_3). One C_q signal of both diastereoisomers is overlapped. HR-MS (+ESI) *m/z* calculated for $C_{17}H_{16}I$ $[M-OH]^+$ 347.0297, found 347.0291.

Compound 4-(1-hydroxy-3-methyl-3-phenylcyclobutyl)benzonitrile (1g) was

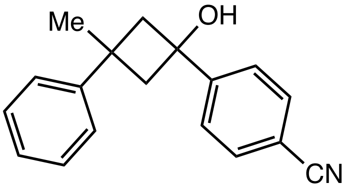

obtained from 4-iodobenzonitrile (939 mg, 4.10 mmol), isopropylmagnesium chloride (3.4 mL, 4.20 mmol) and 3-methyl-3-phenylcyclobutan-1-one (624 mg, 4.00 mmol) following the representative procedure **A** as a white solid by precipitation after 6 hours of reaction time (641 mg, 2.43 mmol, 61%). The product was obtained and characterized as a mixture of diastereoisomers in 1:0.86 ratio. IR (cm^{-1}): ν 3376, 3295, 2975, 2934, 2225, 1608, 1492, 1416, 1396, 1272, 1179, 1116, 1092, 1027, 895, 855, 831, 763, 703, 567, 543. ^{1}H-NMR (300 MHz, $CDCl_3$): δ 7.72 – 7.64 (m, 3.6 H), 7.57 – 7.53 (m, 2.0 H), 7.44 – 7.30 (m, 8.0 H), 7.25 – 7.16 (m, 4.2 H), 2.99 – 2.91 (m, 5.7 H), 2.65

– 2.61 (m, 2.1 H), 2.17 (s, 1 H), 2.01 (s, 0.7 H), 1.71 (s, 3.0 H), 1.32 (s, 2.6 H). $^{13}C\{^1H\}$-NMR (75.45 MHz, $CDCl_3$): δ 152.4 (s, C_q), 151.3 (s, C_q), 150.8 (s, C_q), 150.7 (s, C_q), 132.4 (s, CH), 132.2 (s, CH), 128.5 (s, CH), 128.4 (s, CH), 126.4 (s, CH), 125.8 (s, CH), 125.6 (s, CH), 125.6 (s, CH), 125.3 (s, CH), 125.0 (s, CH), 118.8 (s, C_q), 118.7 (s, C_q), 111.2 (s, C_q), 110.7 (s, C_q), 72.5 (s, C_q), 72.4 (s, C_q), 49.3 (s, CH_2), 49.0 (s, CH_2), 36.0 (s, C_q), 34.9 (s, C_q), 33.1 (s, CH_3), 31.9 (s, CH_3). GC-MS *m/z* calculated for $C_{18}H_{17}NO$ $[M]^+$ 263.1300, found 263.1289.

Compound methyl 4-(1-hydroxy-3-methyl-3-phenylcyclobutyl)benzoate

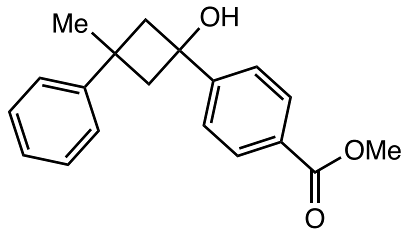

(1h) was obtained from methyl 4-iodobenzoate (1.376 g, 5.25 mmol), isopropylmagnesium chloride (3.0 mL, 5.35 mmol) and 3-methyl-3-phenylcyclobutan-1-one (0.780 g, 5.00 mmol) following the representative procedure **A** as a white solid by precipitation after 5.5 hours of reaction (0.658 g, 2.22 mmol, 44%). The product was isolated and characterized as a mixture of diastereoisomers in 1:0.76 ratio. IR (cm^{-1}): ν 3494, 3314, 2953, 1719, 1692, 1605, 1433, 1273, 1096, 1014, 768, 705. ^1H-NMR (400 MHz, $CDCl_3$): δ 8.09 – 8.06 (m, 2.0 H), 7.95 – 7.92 (m, 1.5 H), 7.63 – 7.61 (m, 2.0 H), 7.39 – 7.29 (m, 7.4 H), 7.23 – 7.15 (m, 3.6 H), 3.93 (s, 3.0 H), 3.88 (s, 2.3 H), 3.01 – 2.89 (m, 5.7 H), 2.65 – 2.61 (m, 1.7 H), 2.13 (s, 0.7 H), 1.99 (s, 1 H), 1.72 (s, 2.3 H), 1.29 (s, 3 H). $^{13}C\{^1H\}$-NMR (100.81 MHz, $CDCl_3$): δ 166.8 (s, C_q), 166.8 (s, C_q), 152.2 (s, C_q), 151.2 (s, C_q), 151.2 (s, C_q), 151.1 (s, C_q), 129.9 (s, CH), 129.7 (s, CH), 129.2 (s, C_q), 128.7 (s, C_q), 128.4 (s, CH), 128.3 (s, CH), 125.7 (s, CH), 125.6 (s, CH), 125.5 (s, CH), 125.2 (s, CH), 125.1 (s, CH), 124.8 (s, CH), 72.7 (s, C_q), 72.4 (s, C_q), 52.1 (s, CH_3), 52.0 (s, CH_3), 49.1 (s, CH_2), 48.7 (s, CH_2), 36.0 (s, C_q), 34.6 (s, C_q), 32.9 (s, CH_3), 31.6 (s, CH_3). GC-MS *m/z* calculated for $C_{19}H_{20}O_3$ $[M]^+$ 296.1412, found 296.1409.

Compound 3-methyl-3-phenyl-1-(pyridin-3-yl)cyclobutan-1-ol (1i) was

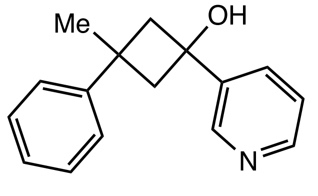

obtained from 3-bromopyridine (0.30 mL, 3.30 mmol), isopropylmagnesium chloride (1.8 mL, 3.30 mmol) and 3-methyl-3-phenylcyclobutan-1-one (481 mg, 3.00 mmol) following a modification of the representative procedure **A** (isopropylmagnesium chloride was added at 0ºC and the mixture was stirred 3 hours at RT) as a white solid after 5 hours of reaction time. The

diastereoisomer **1i** was obtained pure by precipitation (220 mg, 0.90 mmol, 30%). The mother liquors arising from the precipitation were concentrated, and the residue was purified by flash column chromatography using *n*-hexane and EtOAc as eluents to obtain a mixture of diastereoisomers where the major isomer is **1i'** in 1:0.29 ratio (179 mg, 0.75 mmol, 25%). Characterization data of the diastereoisomer **1i** IR (cm^{-1}): ν 3166, 2966, 1425, 1245, 1218, 1124, 1049, 1026, 822, 764, 701, 555. ^{1}H-NMR (400 MHz, $CDCl_3$): δ 8.78 (d, $^4J_{HH}$ = 1.94 Hz, 1 H), 8.51 (dd, $^3J_{HH}$ = 4.88 Hz, $^4J_{HH}$ = 1.39 Hz, 1 H), 7.86 (dt, $^3J_{HH}$ = 7.97 Hz, $^4J_{HH}$ = 1.90 Hz, 1 H), 7.38 – 7.29 (m, 5 H), 7.23 – 7.19 (m, 1 H), 2.97 – 2.90 (m, 4 H), 2.54 (s, 1 H), 1.30 (s, 3 H). $^{13}C\{^1H\}$-NMR (100.81 MHz, $CDCl_3$): δ 151.1 (s, C_q), 148.5 (s, CH), 147.6 (s, CH), 141.6 (s, C_q), 133.5 (s, CH), 128.4 (s, CH), 125.7 (s, CH), 125.2 (s, CH), 123.3 (s, CH), 71.0 (s, C_q), 49.0 (s, CH_2), 34.7 (s, C_q), 31.7 (s, CH_3). HR-MS (+ESI) *m/z* calculated for $C_{16}H_{18}NO$ $[M+H]^+$ 240.1388, found 240.1389. Selected NMR signals of diastereoisomer **1i'** extracted from the mixture of diastereoisomers: ^{1}H-NMR (300 MHz, $CDCl_3$): 8.55 – 8.54 (m, 1 H), 8.38 (dd, $^3J_{HH}$ = 4.76 Hz, $^4J_{HH}$ = 1.66 Hz, 1 H), 7.62 – 7.58 (m, 1 H), 2.68 – 2.63 (m, 2 H), 1.72 (s, 3 H). $^{13}C\{^1H\}$-NMR (100.81 MHz, $CDCl_3$): δ 151.0 (s, C_q), 147.8 (s, CH), 146.6 (s, CH), 132.9 (s, CH), 128.3 (s, CH), 125.5 (s, CH), 125.0 (s, CH), 123.1 (s, CH), 71.1 (s, C_q), 48.6 (s, CH_2), 36.2 (s, C_q), 33.0 (s, CH_3).

Compound 1-mesityl-3-methyl-3-phenylcyclobutan-1-ol (1l) was obtained

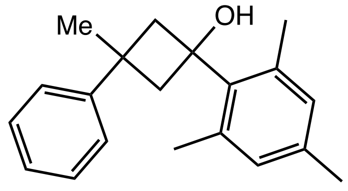

and characterized as a mixture of diastereoisomers in 1:0.42 ratio following the procedure described above. IR (cm^{-1}): ν 3552, 3439, 2927, 1604, 1492, 1443, 1375, 1303, 1194, 1166, 1089, 1028, 988, 956, 907, 850, 762, 730, 702, 580, 549. ^{1}H-NMR (300 MHz, $CDCl_3$): δ 7.49 – 7.46 (m, 0.8 H), 7.41 – 7.36 (m, 1.1 H), 7.28 – 7.23 (m, 2.5 H), 7.16 – 7.08 (m, 2.9 H), 6.82 – 6.81 (m, 0.9 H), 6.78 – 6.76 (m, 2.2 H), 3.21 – 3.16 (m, 0.9 H), 3.09 – 3.04 (m, 1.9 H), 2.96 – 2.91 (m, 0.9 H), 2.84 – 2.78 (m, 1.9 H), 2.37 (s, 2.3 H), 2.35 (s, 5.5 H), 2.25 (s, 1.3 H), 2.20 (m, 4.7 H), 1.98 (s, 0.8 H), 1.72 (s, 3.0 H), 1.35 (s, 1.3 H). $^{13}C\{^1H\}$-NMR (100.81 MHz, $CDCl_3$): δ 152.6 (s, C_q), 150.1 (s, C_q), 140.1 (s, C_q), 136.7 (s, C_q), 136.6 (s, C_q), 136.2 (s, C_q), 130.5 (s, CH), 129.1 (s, CH), 128.5 (s, CH), 128.1 (s, CH), 125.9 (s, CH), 125.6 (s, CH), 125.6 (s, CH), 125.1 (s, CH), 124.8 (s, CH), 76.2 (s, C_q), 75.8 (s, C_q), 52.4 (s, CH_2), 50.9 (s, CH_2), 37.8 (s, C_q),

37.1 (s, C_q), 34.2 (s, CH_3), 31.3 (s, CH_3), 21.6 (s, CH_3), 21.5 (s, CH_3), 20.6 (s, CH_3), 20.5 (s, CH_3), 15.8 (s, CH_3). GC-MS *m/z* calculated for $C_{20}H_{24}O$ $[M]^+$ 280.1827, found 280.1825.

Compound 1-(4-(hydroxymethyl)phenyl)-3-methyl-3-phenylcyclobutan-1-ol

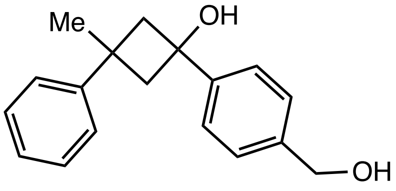

(4) was obtained and characterized as a mixture of diastereoisomers in 1:0.83 ratio following the procedure described above. IR (cm^{-1}): ν 3217, 2965, 2925, 2862, 1493, 1414, 1170, 1011, 905, 763, 732, 696, 542. ^{1}H-NMR (300 MHz, $CDCl_3$): δ 7.56 – 7.53 (m, 1.6 H), 7.41 – 7.25 (m, 12.0 H), 7.22 – 7.12 (m, 4.0 H), 4.70 (s, 1.7 H), 4.62 (s, 2 H), 3.01 – 2.86 (m, 5.4 H), 2.64 – 2.58 (m, 2.1 H), 2.08 (s, 1 H), 1.94 (s, 0.8 H), 1.80 (s, 1 H), 1.71 (s, 3 H), 1.61 (s, 1.5 H), 1.26 (s, 2.4 H). $^{13}C\{^1H\}$-NMR (75.45 MHz, $CDCl_3$): δ 151.5 (s, C_q), 146.9 (s, C_q), 145.8 (s, C_q), 140.0 (s, C_q), 139.6 (s, C_q), 128.3 (s, CH), 128.2 (s, CH), 127.2 (s, CH), 127.0 (s, CH), 126.0 (s, CH), 125.5 (s, CH), 125.3 (s, CH), 125.2 (s, CH), 125.1 (s, CH), 124.9 (s, CH), 72.8 (s, C_q), 72.3 (s, C_q), 65.0 (s, CH_2), 64.9 (s, CH_2), 48.8 (s, CH_2), 48.5 (s, CH_2), 36.0 (s, C_q), 34.3 (s, C_q), 32.7 (s, CH_3), 31.4 (s, CH_3). One C_q signal of both diastereoisomers is overlapped. HR-MS (+ESI) *m/z* calculated for $C_{18}H_{19}O$ $[M\text{-}OH]^+$ 251.1436, found 251.1430.

Compound 3-methyl-1-(4-(phenoxymethyl)phenyl)-3-phenylcyclobutan-1-ol

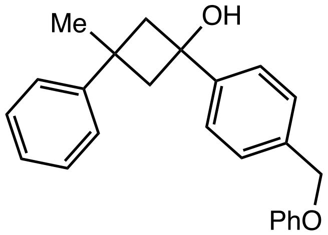

(5) was isolated and characterized as a mixture of diastereoisomers in aprox. 1:1 ratio following the procedure described above. IR (cm^{-1}): ν 3381, 3056, 3024, 2923, 2860, 1597, 1493, 1273, 1167, 1030, 1011, 869, 814, 751, 692, 544, 513. ^{1}H-NMR (300 MHz, $CDCl_3$): δ 7.59 – 7.56 (m, 2 H), 7.50 – 7.47 (m, 2 H), 7.38 – 7.17 (m, 18 H), 7.01 – 6.92 (m, 6 H), 5.08 (s, 2 H), 5.01 (s, 2 H), 3.01 – 2.87 (m, 6 H), 2.64 – 2.60 (m, 2 H), 2.01 (s, 1 H), 1.88 (s, 1 H), 1.72 (s, 3 H), 1.27 (s, 3 H). $^{13}C\{^1H\}$-NMR (75.45 MHz, $CDCl_3$): δ 158.7 (s, C_q), 158.7 (s, C_q), 151.5 (s, C_q), 147.2 (s, C_q), 146.1 (s, C_q), 136.3 (s, C_q), 135.8 (s, C_q), 129.6 (s, CH), 129.5 (s, CH), 128.3 (s, CH), 128.2 (s, CH), 127.8 (s, CH), 127.5 (s, CH), 126.0 (s, CH), 125.5 (s, CH), 125.3 (s, CH), 125.3 (s, CH), 125.1 (s, CH), 125.0 (s, CH), 121.0 (s, CH), 120.9

(s, CH), 114.8 (s, CH), 114.8 (s, CH), 72.8 (s, C_q), 72.3 (s, C_q), 69.9 (s, CH_2), 69.5 (s, CH_2), 48.9 (s, CH_2), 48.5 (s, CH_2), 36.0 (s, C_q), 34.4 (s, C_q), 32.7 (s, CH_3), 31.5 (s, CH_3). One C_q signal of both diastereoisomers is overlapped. HR-MS (+ESI) *m/z* calculated for $C_{24}H_{23}O$ $[M-OH]^+$ 327.1749, found 327.1747.

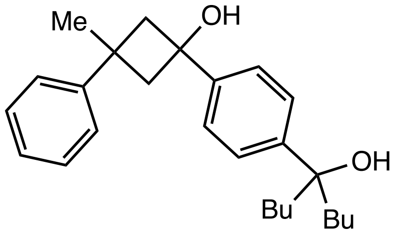

Compound 1-(4-(5-hydroxynonan-5-yl)phenyl)-3-methyl-3-phenylcyclobutan-1-ol (7) was isolated and characterized as a mixture of diastereoisomers in aprox. 1:0.75 ratio following the procedure described above. IR (cm^{-1}): ν 3438, 3342, 2949, 2928, 2864, 1491, 1442, 1402, 1281, 1238, 1118, 1045, 1025, 836, 764, 700, 648, 600, 543. ^{1}H-NMR (300 MHz, $CDCl_3$): δ 7.51 – 7.48 (m, 2.3 H), 7.40 – 7.11 (m, 18.4 H), 3.01 – 2.84 (m, 7.4 H), 2.63 – 2.58 (m, 2.0 H), 2.21 (s, 1.0 H), 2.08 (s, 0.9 H), 1.82 – 1.69 (m, 14.3 H), 1.30 – 1.15 (m, 16.8 H), 1.07 – 0.92 (m, 4.9 H), 0.86 – 0.78 (m, 12.7 H). ^{13}C{^{1}H}-NMR (75.45 MHz, $CDCl_3$): δ 151.7 (s, C_q), 145.7 (s, C_q), 145.2 (s, C_q), 145.2 (s, C_q), 144.2 (s, C_q), 128.3 (s, CH), 128.1 (s, C_q), 125.4 (s, CH), 125.3 (s, CH), 125.2 (s, CH), 125.2 (s, CH), 125.1 (s, CH), 124.4 (s, CH), 76.9 (s, C_q), 76.8 (s, C_q), 72.7 (s, C_q), 72.2 (s, C_q), 48.9 (s, CH_2), 48.3 (s, CH_2), 42.6 (s, CH_2), 42.6 (s, CH_2), 36.0 (s, C_q), 34.3 (s, C_q), 32.7 (s, CH_3), 31.4 (s, CH_3), 25.6 (s, CH_2), 25.5 (s, CH_2), 23.0 (s, CH_2), 23.0 (s, CH_2), 14.0 (s, CH_3). Some signals of both diastereoisomers are overlapped. HR-MS (+ESI) *m/z* calculated for $C_{26}H_{35}O$ $[M-OH]^+$ 363.2688, found 363.2688.

Representative procedure and optimization table for two-fold C-C cleavage reaction

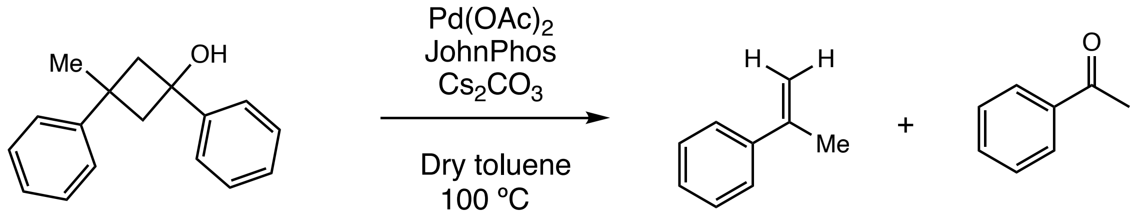

A mixture of cyclobutanol (0.30 mmol, 1 equiv.), $Pd(OAc)_2$, JohnPhos and caesium carbonate (1.1 equiv.) (see the table of the scope of the reaction to check the quantities of catalyst and ligand in each case) in dry toluene (3 mL) under N_2 was heated at 100 ºC (silicon oil bath) in a Carius tube overnight. The mixture was diluted with dichloromethane, filtered over celite, and concentrated in vacuum carefully. The crude mixture was purified by flash column chromatography in silica gel using *n*-hexane and Et_2O as solvents (gradient 0 to 50% Et_2O).

Table S1. Study of reaction parameters.

Changing conditions	Conversion (%)
No ligand	-
No catalyst	-
No base	-
L = PPh_3	-
L = SPhos	67
Base = Et_3N	-
Base = K_2CO_3	70
Air atmosphere	95

* The optimization of the reaction was carried out with the substrate **1a**. The conversion was determined by the measurement of the ^{1}H-NMR of the crude reaction mixture after addition of the internal standard.

Scope of the two-fold C-C cleavage reaction

Table S2. Scope of the two-fold C-C cleavage reaction

Substrate	Pd(OAc) (mol%)	JohnPhos (mol%)	NMR yield (%)	Isolated yield (%)	Internal standard
**1a**	2	4	85	72	4-nitrobenzaldehyde
**1b**	2	4	67	63	4-nitrobenzaldehyde
**1c**	2	4	90	84	4-nitrobenzaldehyde
**1d**	2	4	88	80	1,2-dibromoethane
**1e**	2	4	-	74	-
**1e**[a]	2	4	-	65	-
**1f**	2	4	40	37	1,2-dibromoethane
**1g**	5	10	75	66	1,2-dibromoethane
**1h**	2	4	79	64	4-nitrobenzaldehyde
**1i**	2	4	92	78	4-nitrobenzaldehyde
**1n**[b]	10	20	>99/76	83/70	1,2-dibromoethane
**5**	8	16	68	61	1,2-dibromoethane
**7**	5	10	72	65	1,2-dibromoethane

[a] Reaction carried out in larger scale (1.5 mmol of substrate)
[b] NMR and isolated yield for 1,1-diphenylethylene and acetophenone respectively

NMR data and isolated yields of the obtained products formed upon the Pd-catalyzed C–C cleavage process

Acetophenone **2a** was obtained as a colourless liquid (26 mg, 0.22 mmol, 72%) ^{1}H-NMR (300 MHz, $CDCl_3$): δ 7.98 – 7.95 (m, 2 H), 7.57 – 7.44 (m, 3 H), 2.61 (s, 3 H). The signals match with those described in the literature.[4]

Acetophenone **2b** was obtained as a colourless liquid (25 mg, 0.19 mmol, 63%) ^{1}H-NMR (300 MHz, $CDCl_3$): δ 7.87 – 7.84 (m, 2 H), 7.27 – 7.24 (m, 2 H) 2.58 (s, 3 H), 2.41 (s, 3 H). The signals match with those described in the literature.[5]

Acetophenone **2c** was obtained as a white solid (38 mg, 0.25 mmol, 84%) ^{1}H-NMR (300 MHz, $CDCl_3$): δ 7.95 – 7.92 (m, 2 H), 6.94 – 6.91 (m, 2 H), 3.86 (s, 3 H), 2.55 (s, 3 H). The signals match with those described in the literature.[6]

Acetophenone **2d** was obtained as a colourless liquid (33 mg, 0.24 mmol, 80%) ^{1}H-NMR (300 MHz, $CDCl_3$): δ 8.01 – 7.96 (m, 2 H), 7.16 – 7.10 (m, 2 H), 2.59 (s, 3 H). $^{19}F\{^{1}H\}$-NMR (282.4 MHz, $CDCl_3$): δ -105.3 (s). The signals match with those described in the literature.[7]

Acetophenone **2e** was obtained as a white solid (44 mg, 0.22 mmol, 74%) ^{1}H-NMR (300 MHz, $CDCl_3$): δ 7.83 – 7.81 (m, 2 H), 7.62 – 7.59 (m, 2 H), 2.58 (s, 3 H). The signals match with those described in the literature.[8] Acetophenone **2e** was also prepared in a larger scale starting from 1.5 mmol of cyclobutanol **1e**, obtaining an isolated amount of 195 mg, 0.98 mmol, 65% yield.

Acetophenone **2f** was obtained as a white solid (27 mg, 0.11 mmol, 37%) ^{1}H-NMR (300 MHz, $CDCl_3$): δ 7.85 – 7.82 (m, 2 H), 7.68 – 7.65 (m, 2 H), 2.58 (s, 3 H). The signals match with those described in the literature.[9]

Acetophenone **2g** was obtained as a white solid (29 mg, 0.20 mmol, 66%) ^{1}H-NMR (300 MHz, $CDCl_3$): δ 8.07 – 8.04 (m, 2 H), 7.80 – 7.77 (m, 2 H), 2.65 (s, 3 H). The signals match with those described in the literature.[10]

Acetophenone **2h** was obtained as a white solid (34 mg, 0.19 mmol, 64%) ^{1}H-NMR (300 MHz, $CDCl_3$): δ 8.14 – 8.11 (m, 2 H), 8.02 – 7.99 (m, 2 H), 3.95 (s, 3 H), 2.65 (s, 3 H). The signals match with those described in the literature.[11]

Product **2i** was obtained as a colourless liquid (28 mg, 0.23 mmol, 78%). ^{1}H-NMR (300 MHz, $CDCl_3$): δ 9.18 (m, 1 H), 8.81 – 8.78 (m, 1 H), 8.26 – 8.23 (m, 1 H), 7.46 – 7.41 (m, 1 H), 2.66 (s, 3 H). The signals match with those described in the literature.[12]

Alkene 1,1-diphenylethylene **3b** was obtained as a colourless liquid (45 mg, 0.25 mmol, 83%) ^{1}H-NMR (300 MHz, $CDCl_3$): δ 7.35 – 7.29 (m, 10 H), 5.45 (s, 2 H). The signals match with the data described in the literature.[13]

Acetophenone **6** was obtained as a white solid (41 mg, 0.18 mmol, 61%) ^{1}H-NMR (300 MHz, $CDCl_3$): δ 7.99 – 7.96 (m, 2 H), 7.54 – 7.51 (m, 2 H), 7.32 – 7.27 (m, 2 H), 6.98 – 6.95 (m, 3 H), 5.13 (s, 2H), 2.61 (s, 3 H). The signals match with those described in the literature.[14]

Acetophenone **8** was obtained as a colourless oil (51 mg, 0.20 mmol, 65%) ^{1}H-NMR (300 MHz, $CDCl_3$): δ 7.94 – 7.92 (m, 2 H), 7.49 – 7.46 (m, 2 H), 2.60 (s, 3 H), 1.86 – 1.75 (m, 4 H), 1.28 – 1.23 (m, 6 H), 1.02 – 0.93 (m, 2 H), 0.85 – 0.80 (m, 6 H). The signals match with those described in the literature.[15]

References

[1] Seiser, T.; Roth, O.A.; Cramer, N. *Angew. Chem. Int. Ed.* **2009**, *48*, 6320-6323.

[2] Brunetti, A.; Pintus, A.; Lombardi, L.; Kovtun, A.; Mascietti, F.; Bruno, F.; Ravera, E.; Melucci, M.; Bertuzzi, G.; Bandini, M. *Chin. J. Chem.* **2023**, *41*, 1333-1340.

[3] Croft, R.A.; Mousseau, J.J.; Choi, C.; Bull, J.A. *Chem. Eur. J.* **2018**, *24*, 818-821.

[4] Wang, S.; Miao, C.; Wang, W.; Lei, Z.; Sun, W. *ChemCatChem* **2014**, *6*, 1612-1616.

[5] Zhang, G.; Han, X.; Luan, Y.; Wang, Y.; Wen, X.; Ding, C. *Chem. Commun.* **2013**, *49*, 7908-7910.

[6] Behrends, M.; Sävmarker, J.; Sjöberg, P.J.R.; Larhed, M. *ACS Catal.* **2011**, *1*, 1455-1459.

[7] Ebule, R.E.; Malhotra, D.; Hammond, G.B.; Xu, B. *Adv. Synth. Catal.* **2016**, *358*, 1478-1481.

[8] Pan, J.; Wang, X.; Zhang, Y.; Buchwald, S.L. *Org. Lett.* **2011**, *13*, 4974-4976.

[9] Liu, W.; Yang, X.; Gao, Y.; Li, C.J. *J. Am. Chem. Soc.* **2017**, *139*, 8621-8627.

[10] Schulz, J.; Císarǒva, I.; Štep̌nick̆a, P. *Organometallics* **2012**, *31*, 729-738.

[11] Jacobson, C.E.; Martinez-Muñoz, N.; Gorin, D.J. *J. Org. Chem.* **2015**, *80*, 7305-7310.

[12] Liu, J.; Zhang, X.; Yi, H.; Liu, C.; Liu, R.; Zhang, H.; Zhuo, K.; Lei, A. *Angew. Chem. Int. Ed.* **2015**, *54*, 1261-1265.

[13] Conner, M.L.; Brown, M.K. *J. Org. Chem.* **2016**, *81*, 8050-8060.

[14] Shields, B.J.; Doyle, A.G. *J. Am. Chem. Soc.* **2016**, *138*, 12719-12722.

[15] Barrios, F.J.; Zhang, X.; Colby, D.A. *Org. Lett.* **2010**, *12*, *23*, 5588-5591.

NMR spectra of the non-previously reported compounds

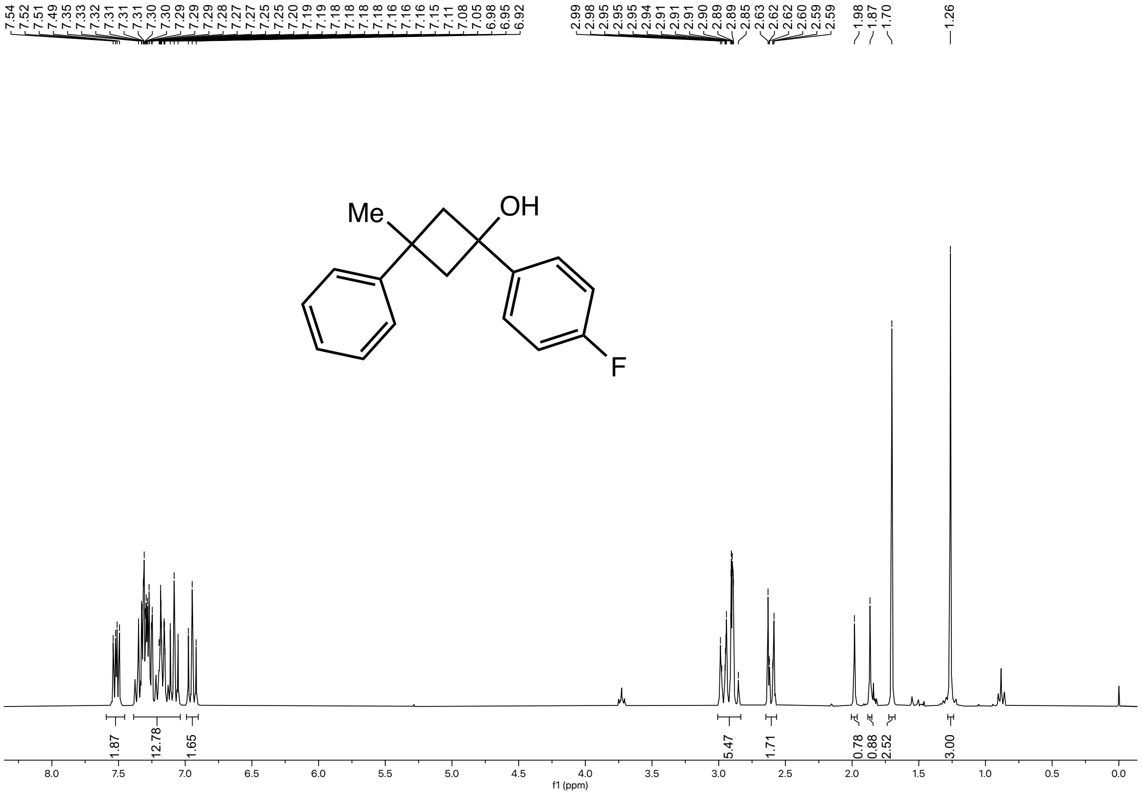

Figure S1. ^{1}H-NMR spectra of 1:0.84 mixture of diastereoisomers of compound **1d** (300 MHz, $CDCl_3$)

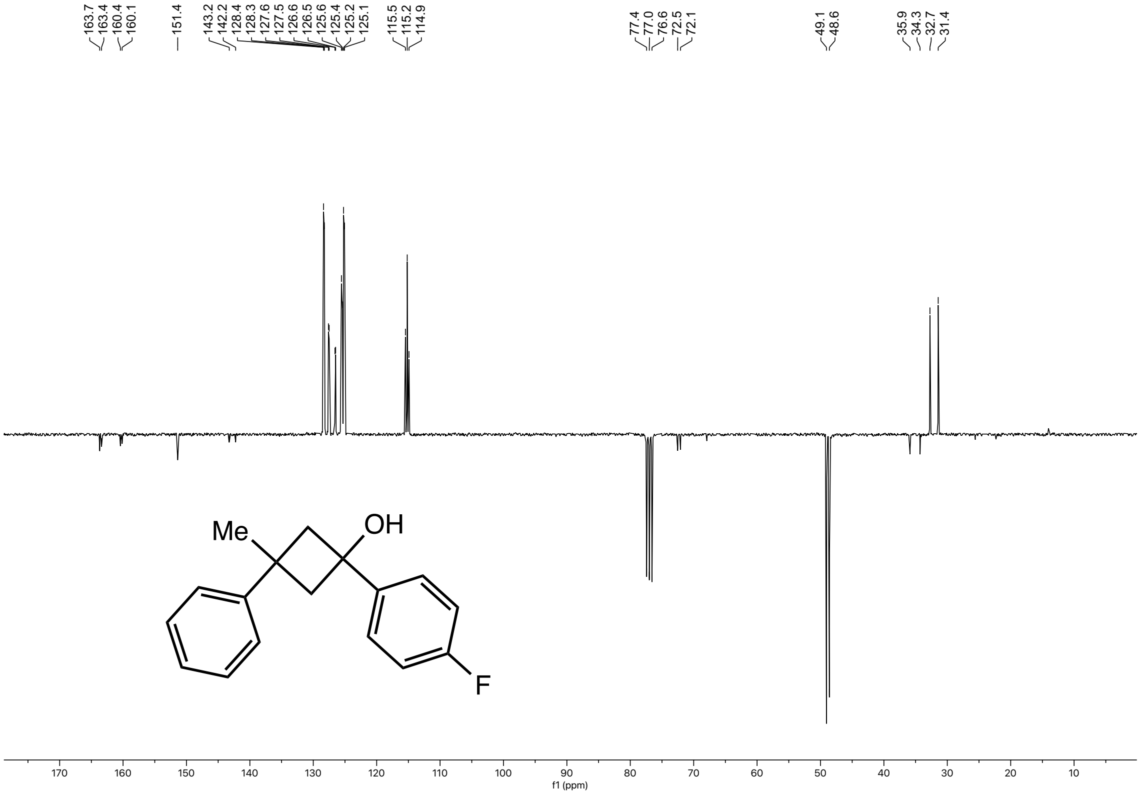

Figure S2. APT $^{13}C\{^{1}H\}$-NMR spectra of 1:0.84 mixture of diastereoisomers of compound **1d** (75.45 MHz, $CDCl_3$)

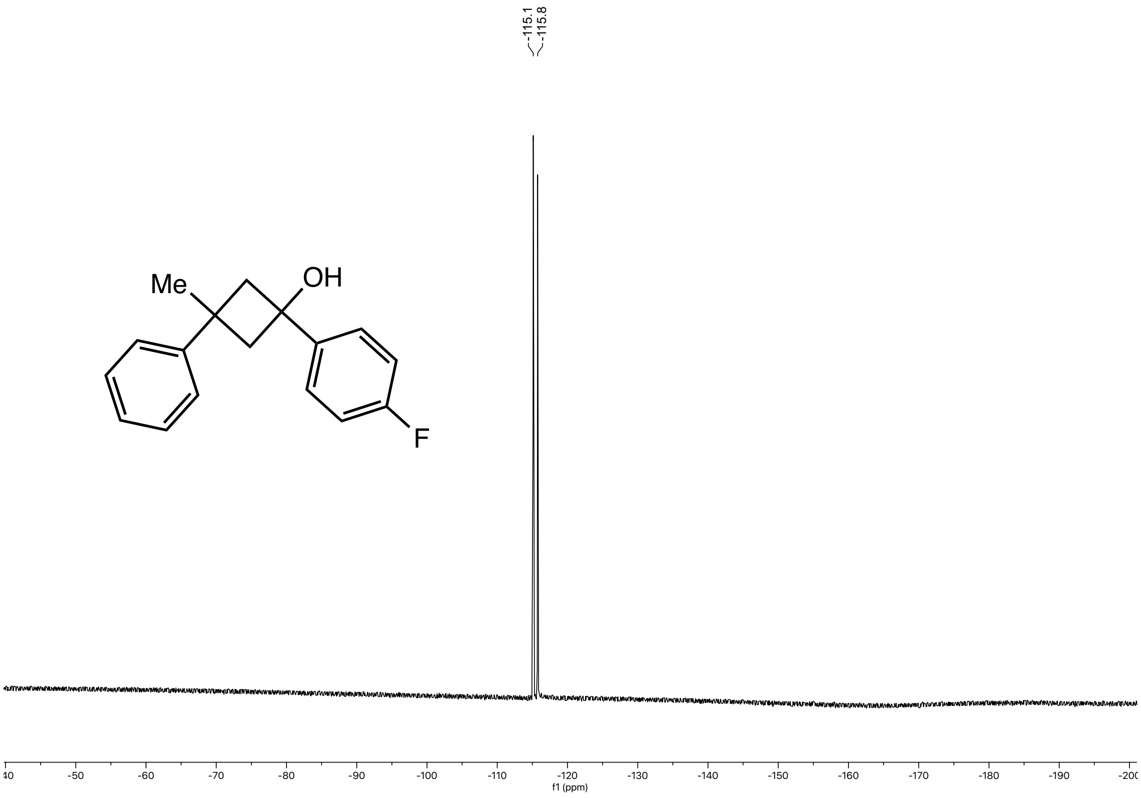

Figure S3. $^{19}F\{^{1}H\}$-NMR spectra of 1:0.84 mixture of diastereoisomers of compound **1d** (282.4 MHz, $CDCl_3$)

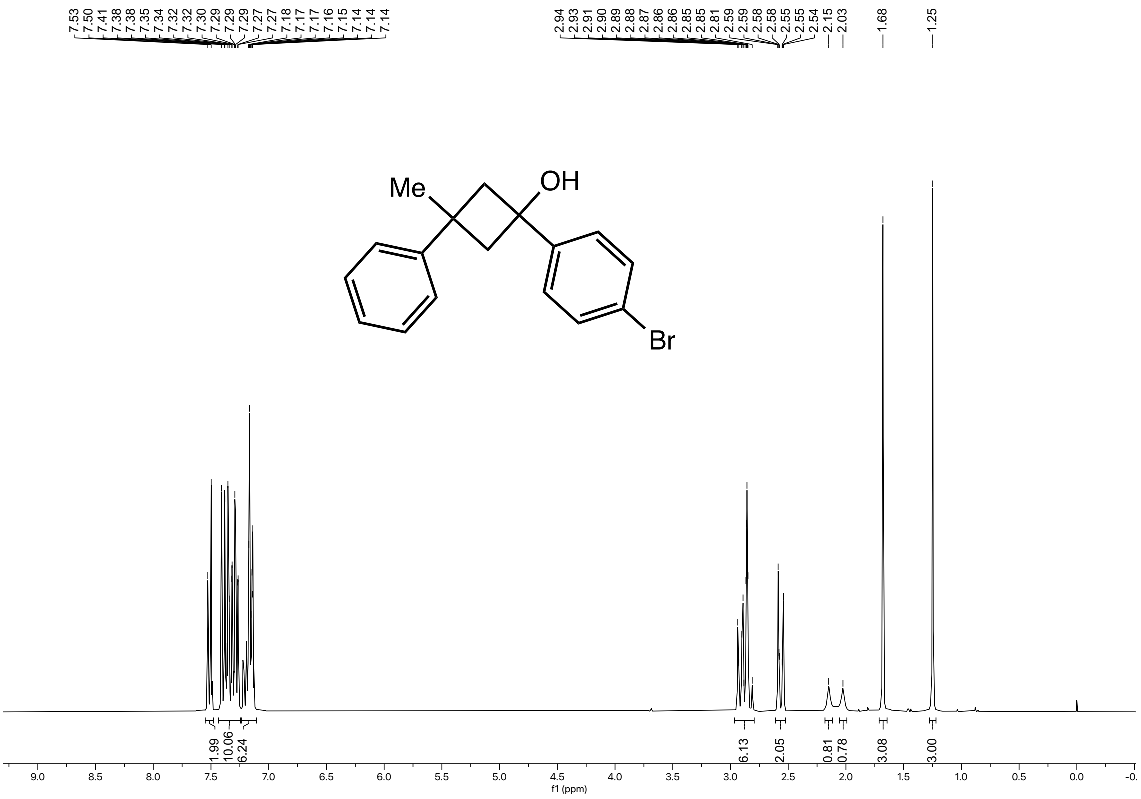

Figure S4. ^{1}H-NMR spectra of 1:1 mixture of diastereoisomers of compound **1e** (300 MHz, $CDCl_3$)

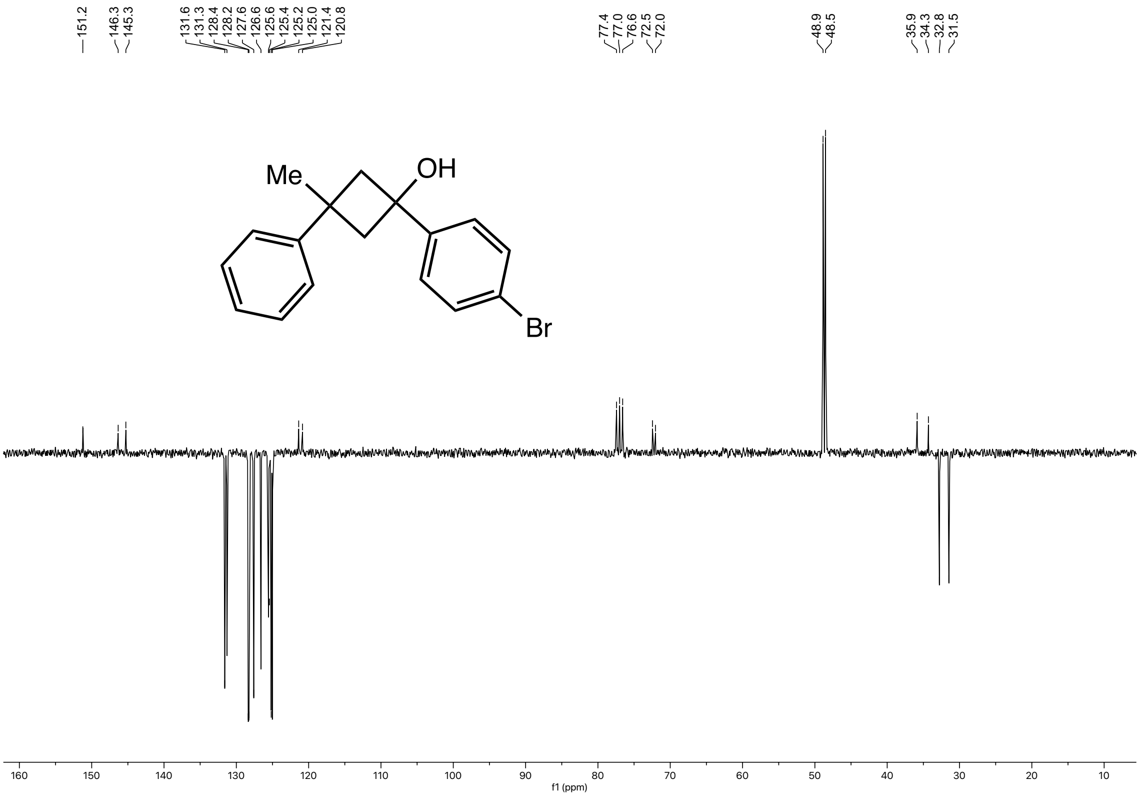

Figure S5. APT $^{13}C\{^{1}H\}$-NMR spectra of 1:1 mixture of diastereoisomers of compound **1e** (75.45 MHz, $CDCl_3$)

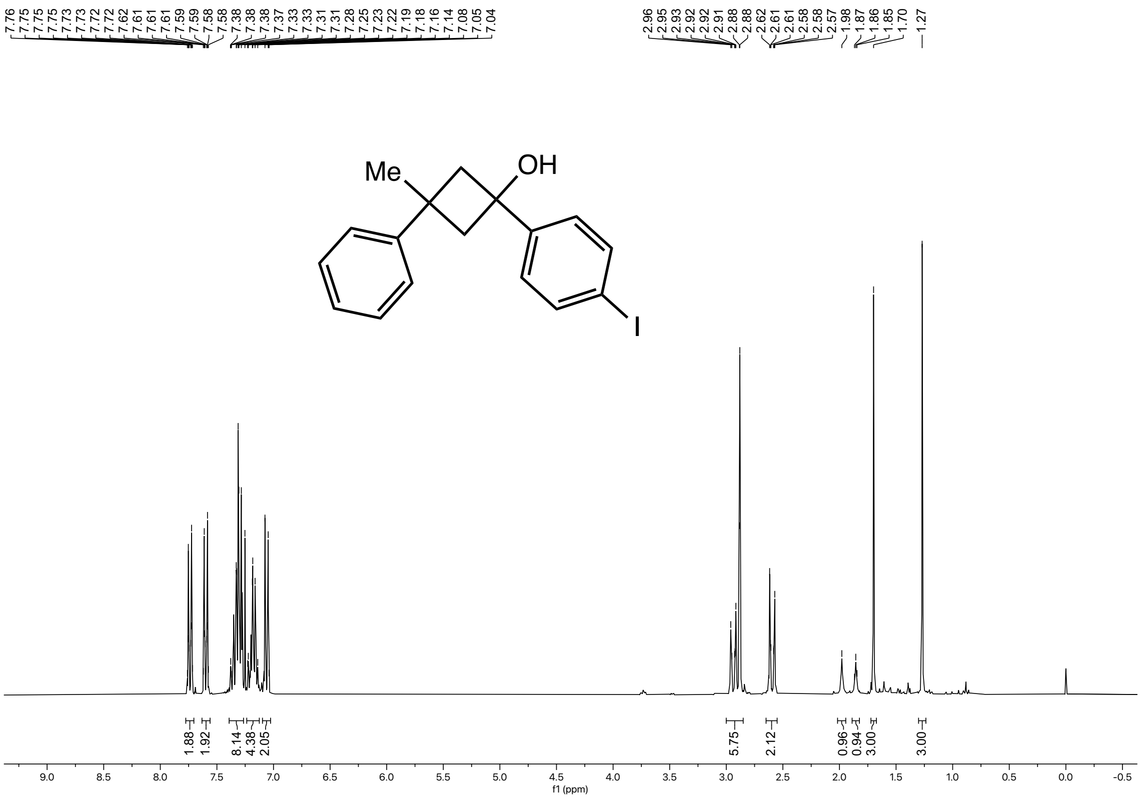

Figure S6. ^{1}H-NMR spectra of 1:1 mixture of diastereoisomers of compound **1f** (300 MHz, $CDCl_3$)

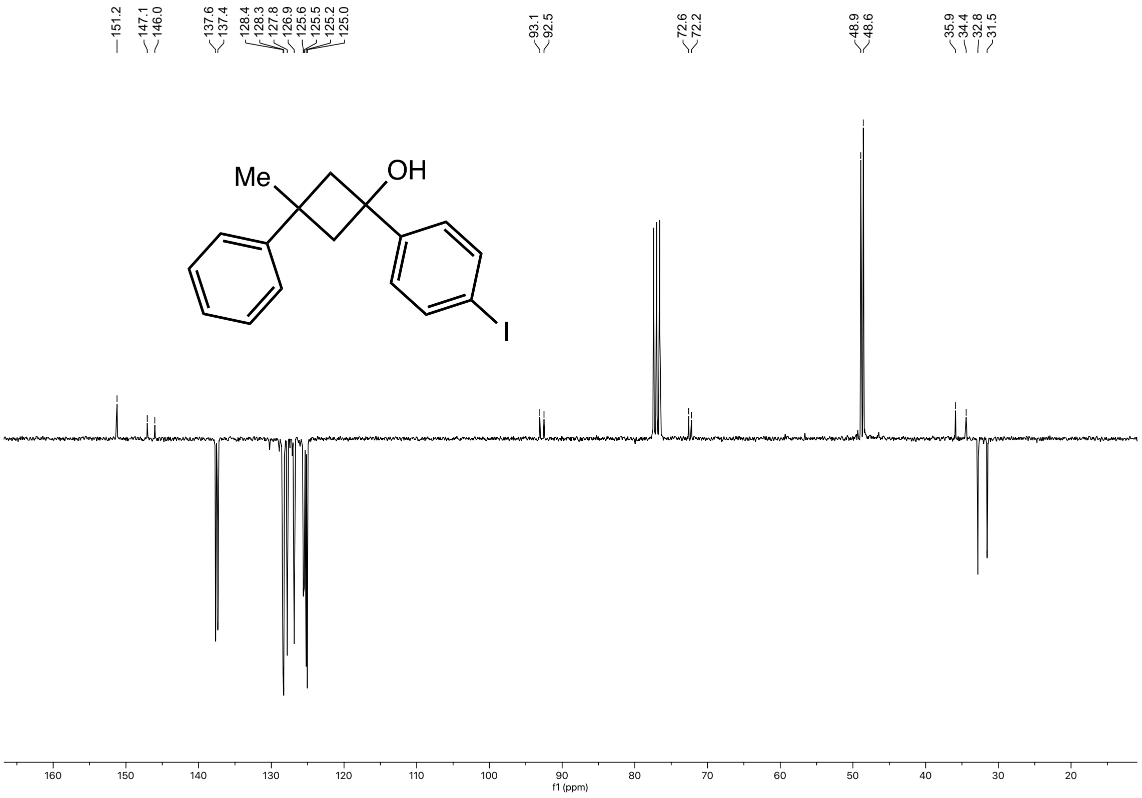

Figure S7. APT $^{13}C\{^{1}H\}$-NMR spectra of 1:1 mixture of diastereoisomers of compound **1f** (75.45 MHz, $CDCl_3$)

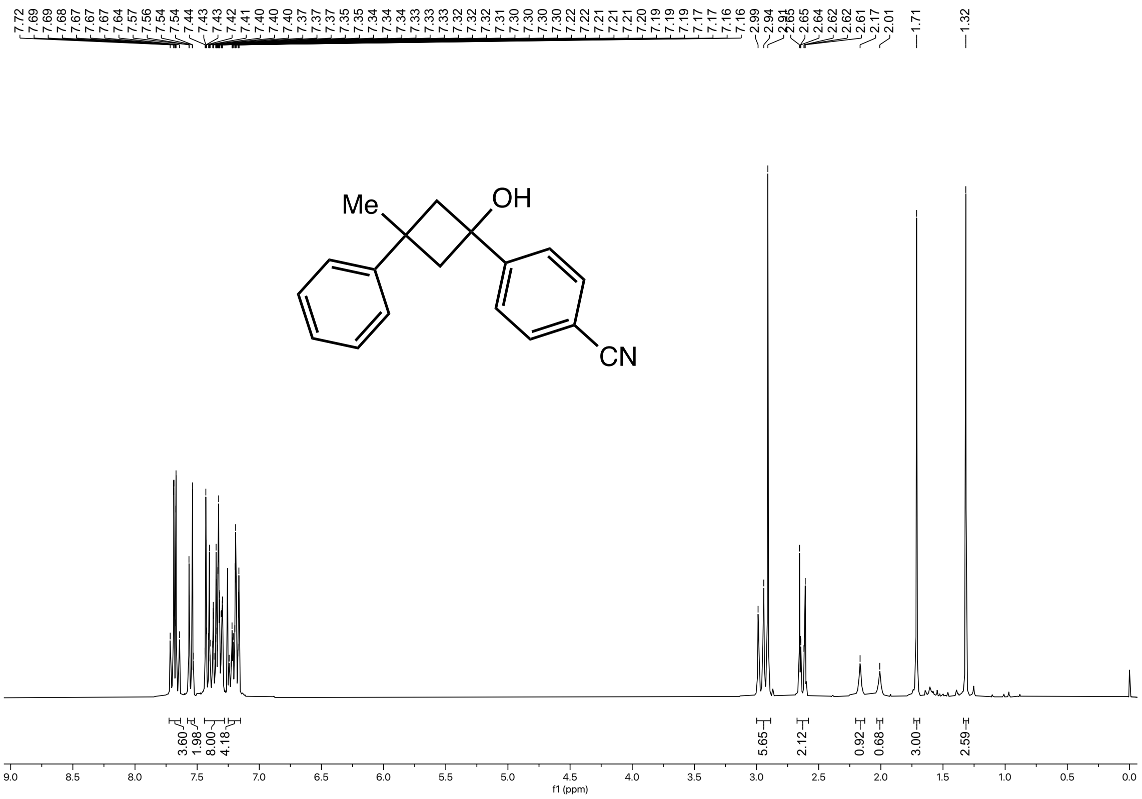

Figure S8. ^{1}H-NMR spectra of 1:0.86 mixture of diastereoisomers of compound **1g** (300 MHz, $CDCl_3$)

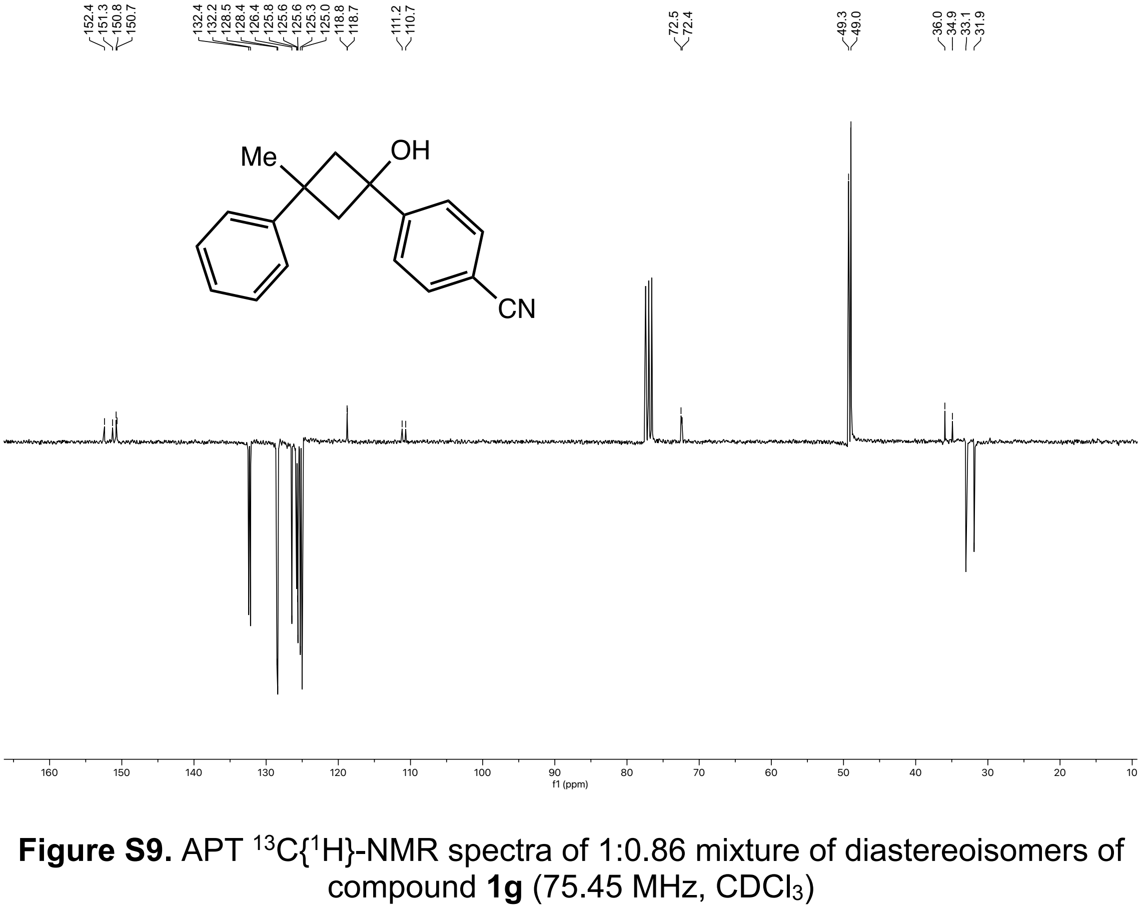

Figure S9. APT $^{13}C\{^{1}H\}$-NMR spectra of 1:0.86 mixture of diastereoisomers of compound **1g** (75.45 MHz, $CDCl_3$)

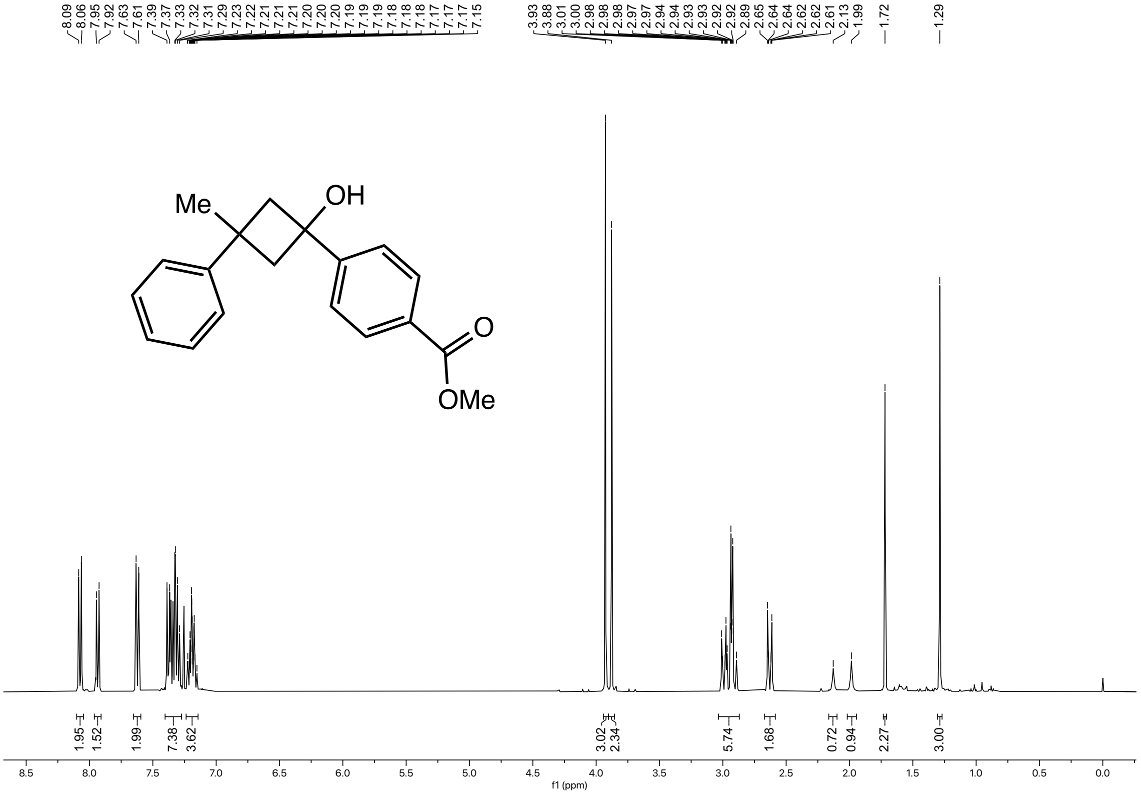

Figure S10. ^{1}H-NMR spectra of 1:0.76 mixture of diastereoisomers of compound **1h** (400 MHz, $CDCl_3$)

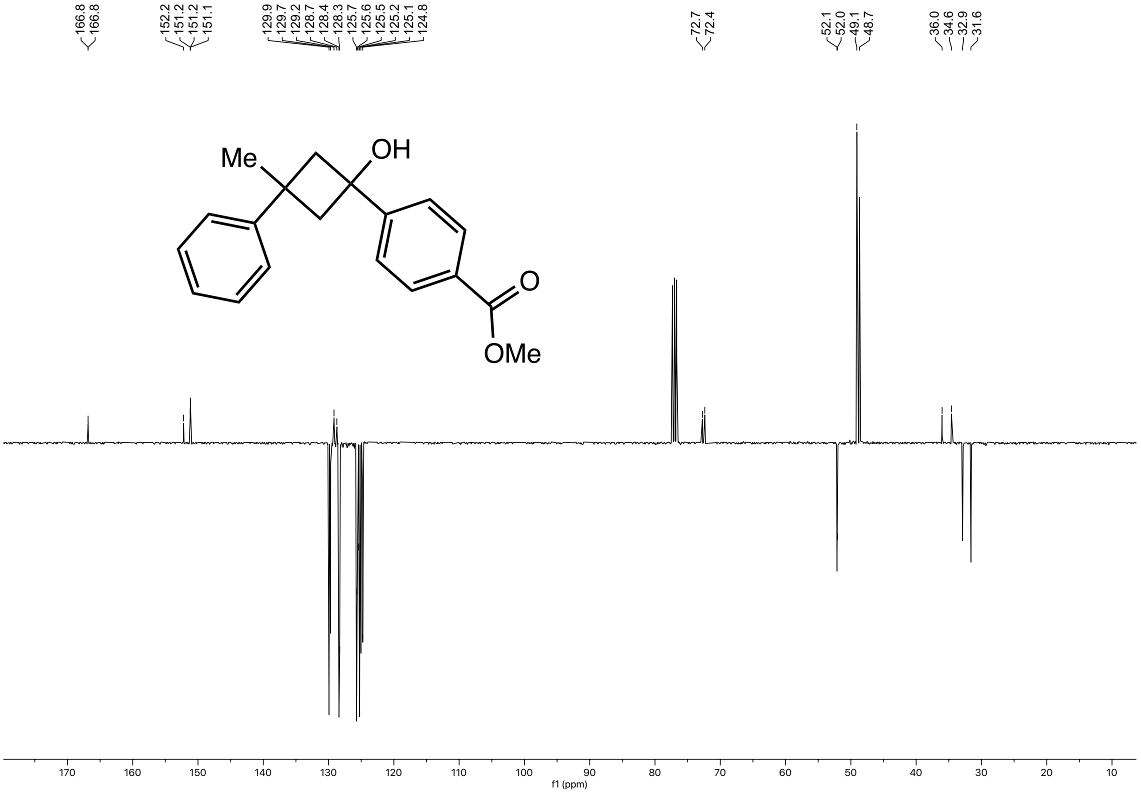

Figure S11. APT $^{13}C\{^{1}H\}$-NMR spectra of 1:0.76 mixture of diastereoisomers of compound **1h** (100.81 MHz, $CDCl_3$)

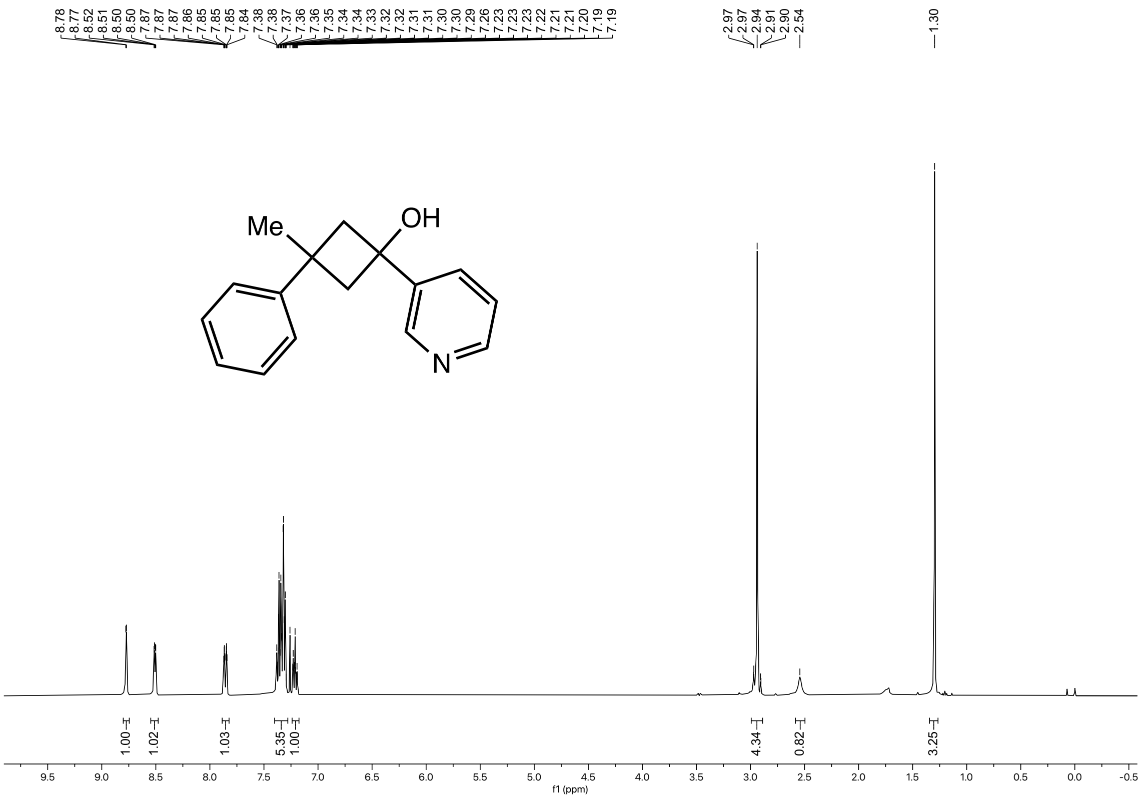

Figure S12. ^{1}H-NMR spectra of compound **1i** (400 MHz, $CDCl_3$)

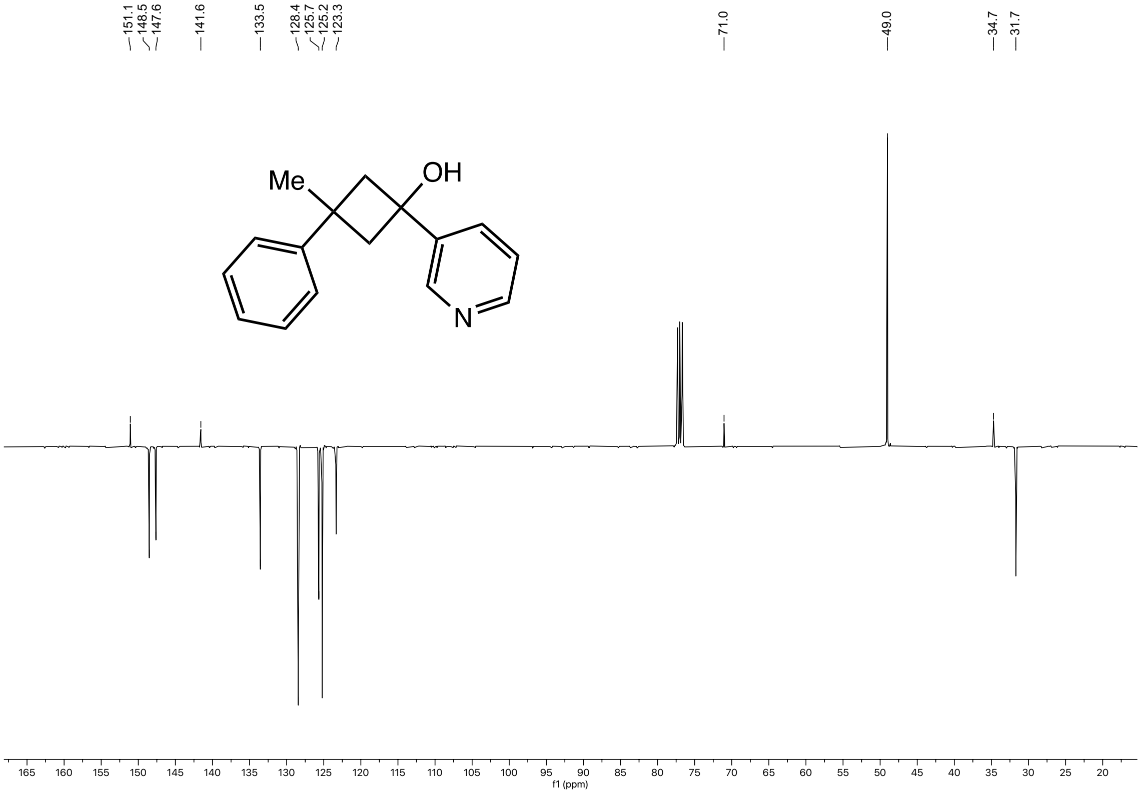

Figure S13. APT $^{13}C\{^{1}H\}$-NMR spectra of compound **1i** (100.81 MHz, $CDCl_3$)

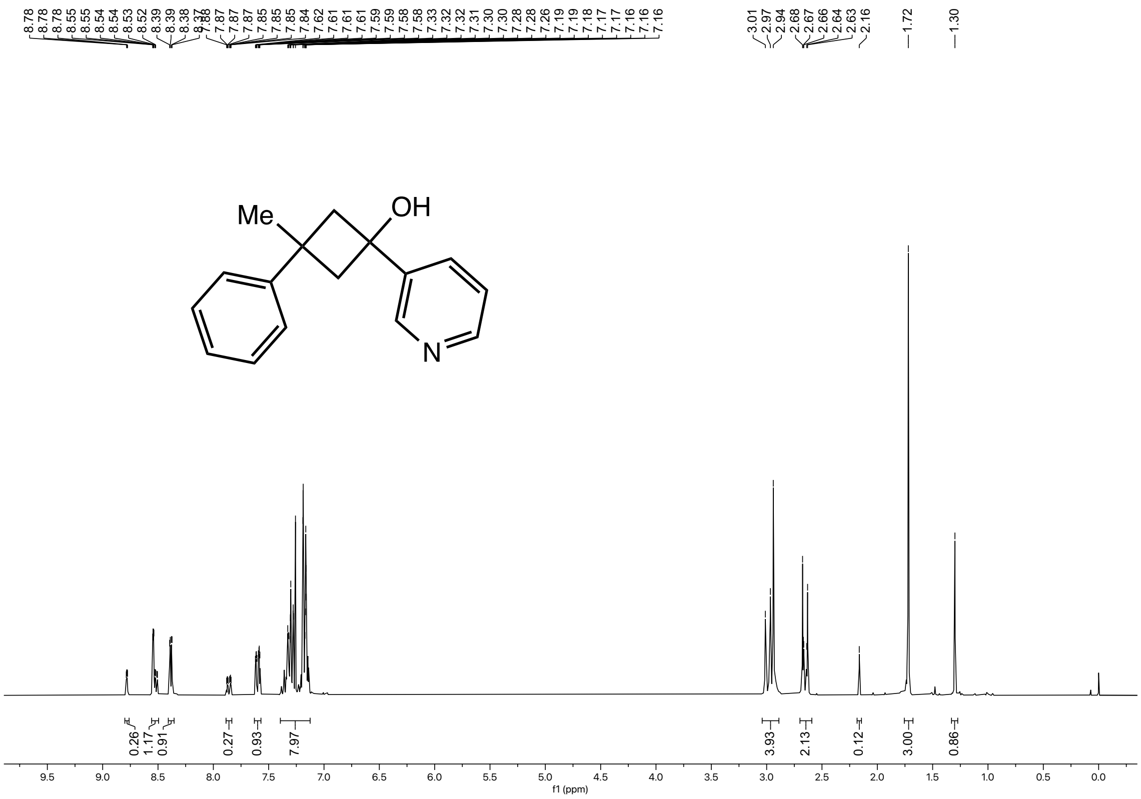

Figure S14. ^{1}H-NMR spectra of mixture of compounds **1i** and **1i’** (300 MHz, $CDCl_3$)

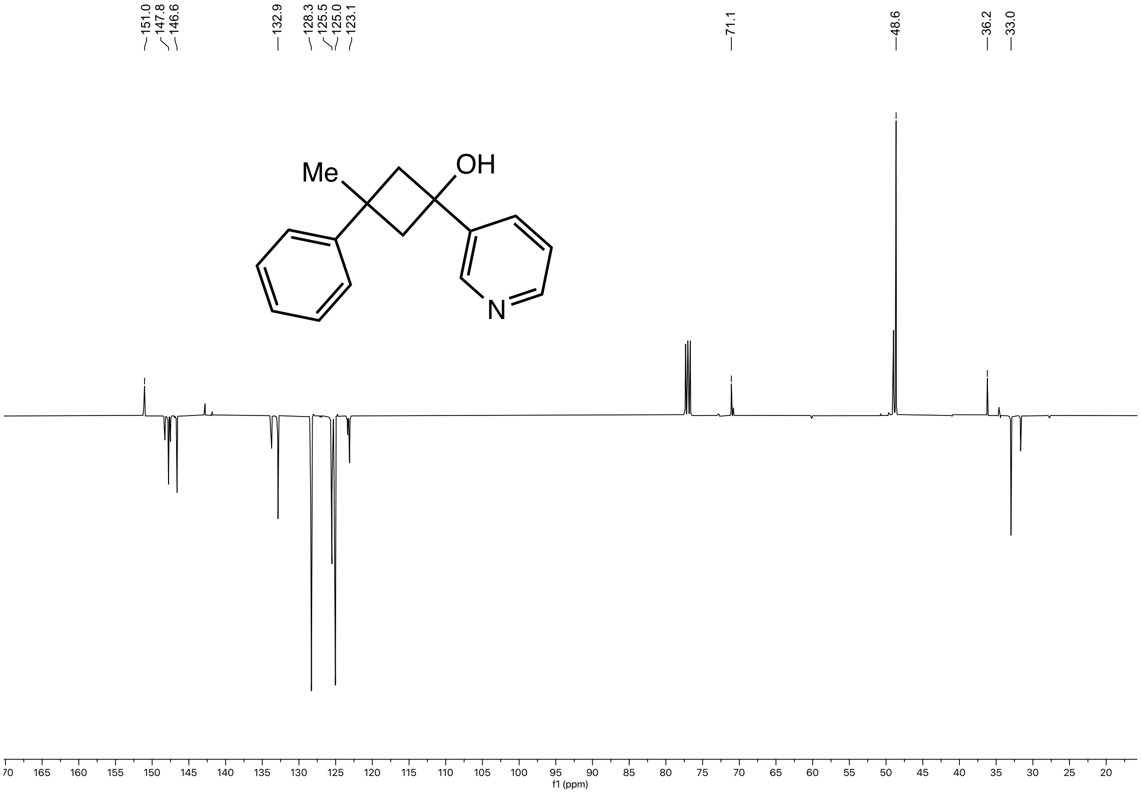

Figure S15. APT $^{13}C\{^{1}H\}$-NMR spectra of mixture of compounds **1i** and **1i’** (100.81 MHz, $CDCl_3$)

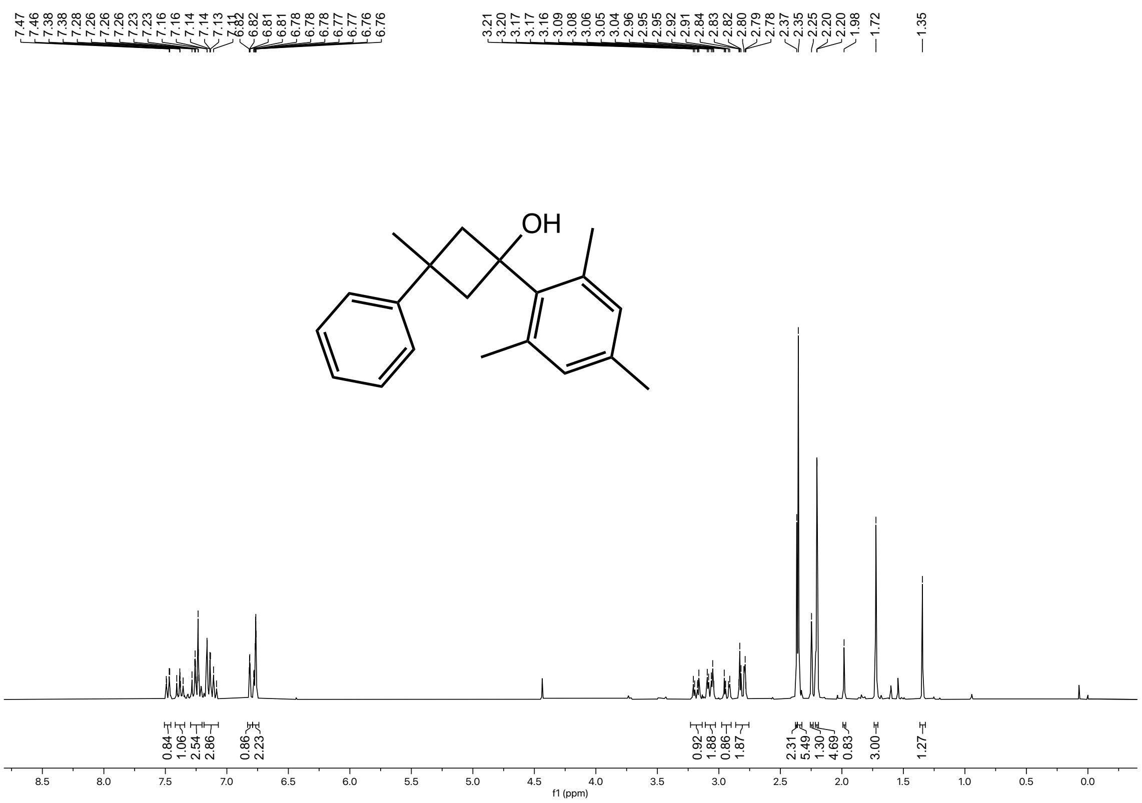

Figure S16. ^{1}H-NMR spectra of 1:0.42 mixture of diastereoisomers of compound **1l** (300 MHz, $CDCl_3$)

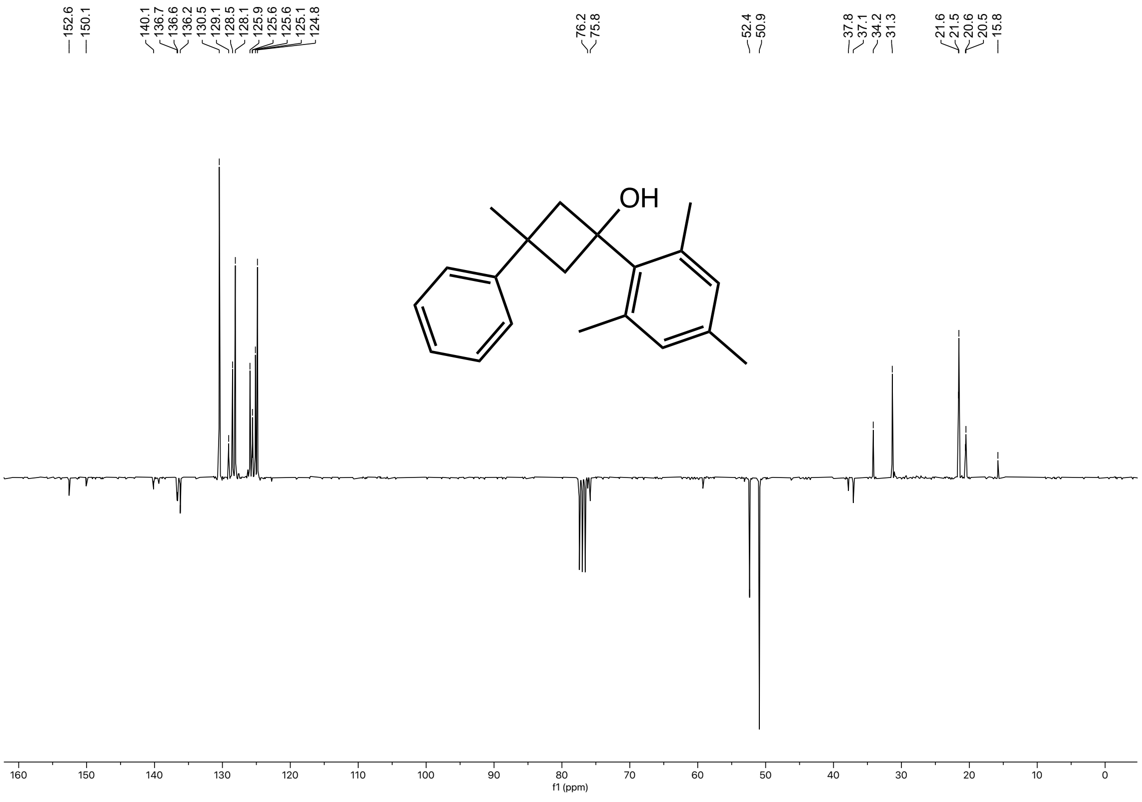

Figure S17. APT ^{13}C{^{1}H}-NMR spectra of 1:0.42 mixture of diastereoisomers of compound **1l** (75.45 MHz, $CDCl_3$)

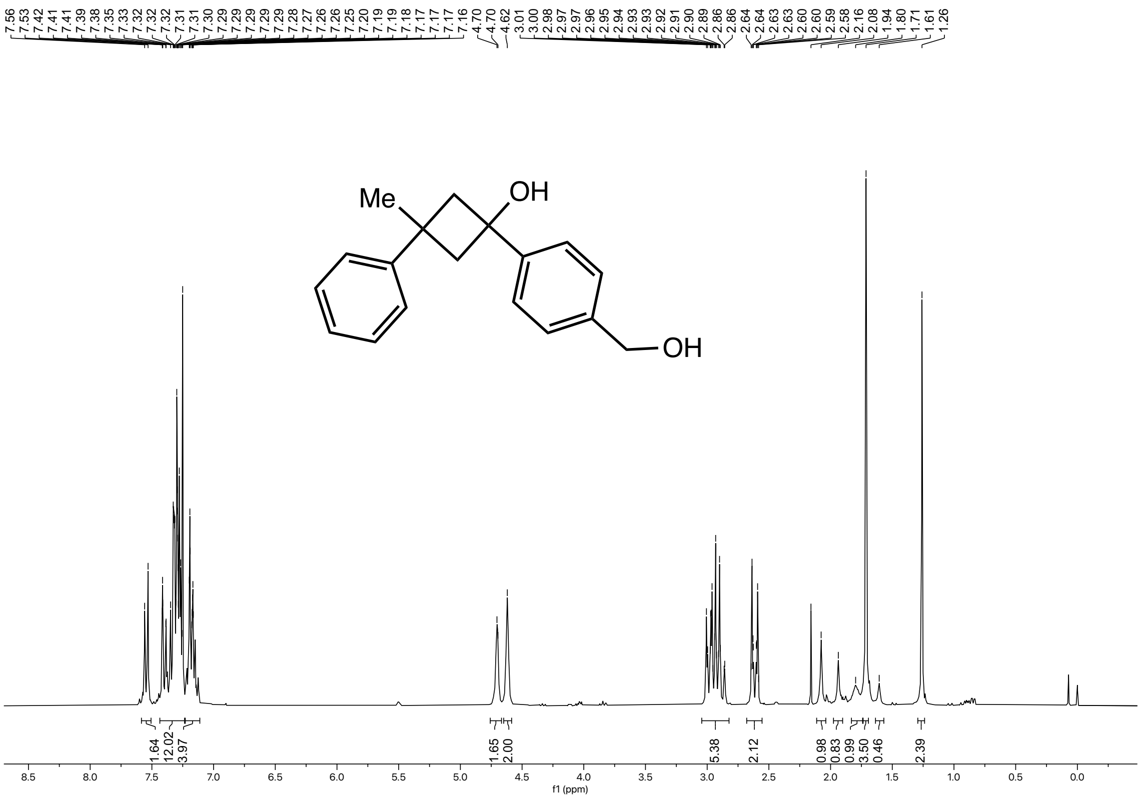

Figure S18. ^{1}H-NMR spectra of 1:0.83 mixture of diastereoisomers of compound **4** (300 MHz, $CDCl_3$)

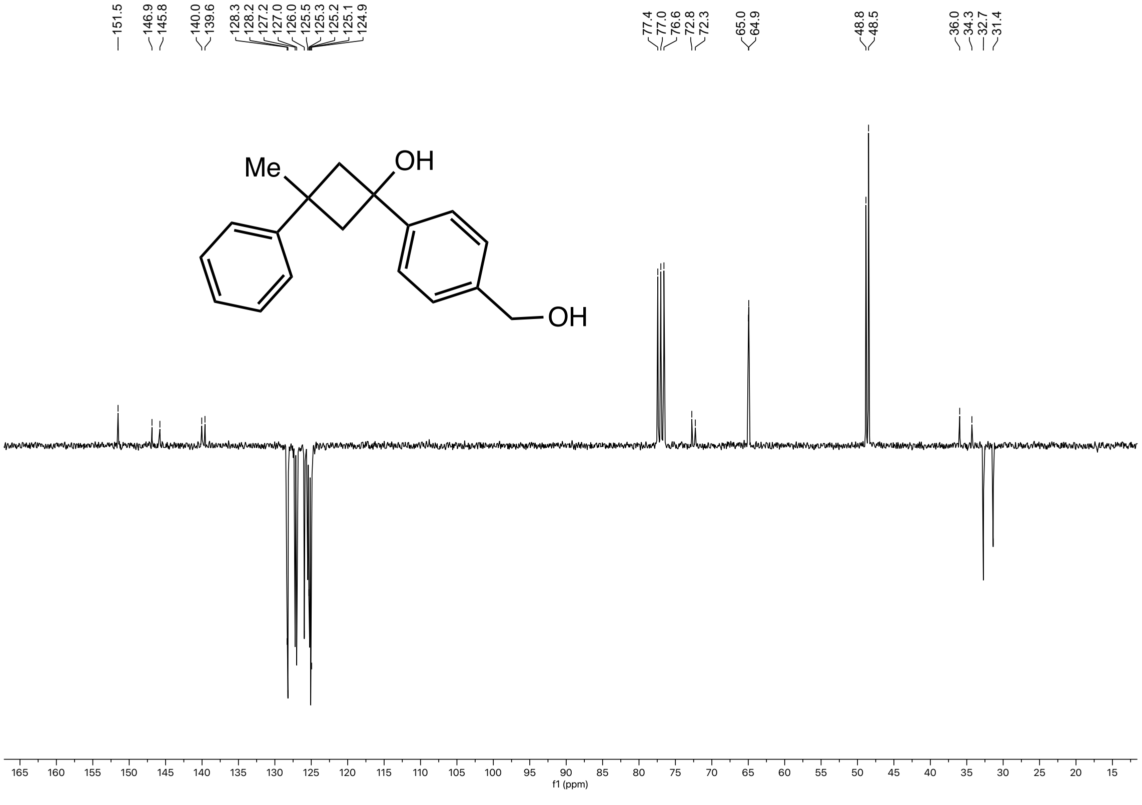

Figure S19. APT $^{13}C\{^{1}H\}$-NMR spectra of 1:0.83 mixture of diastereoisomers of compound **4** (75.45 MHz, $CDCl_3$)

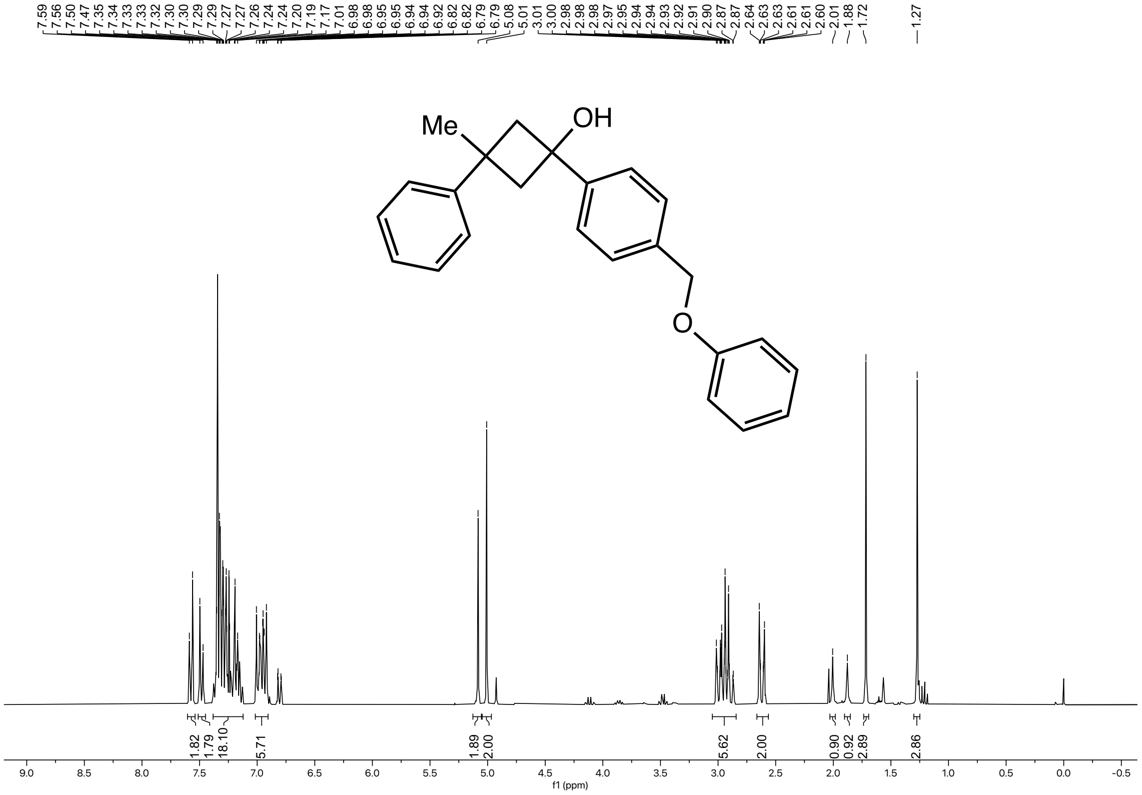

Figure S20. ^{1}H-NMR spectra of 1:1 mixture of diastereoisomers of compound **5** (300 MHz, $CDCl_3$)

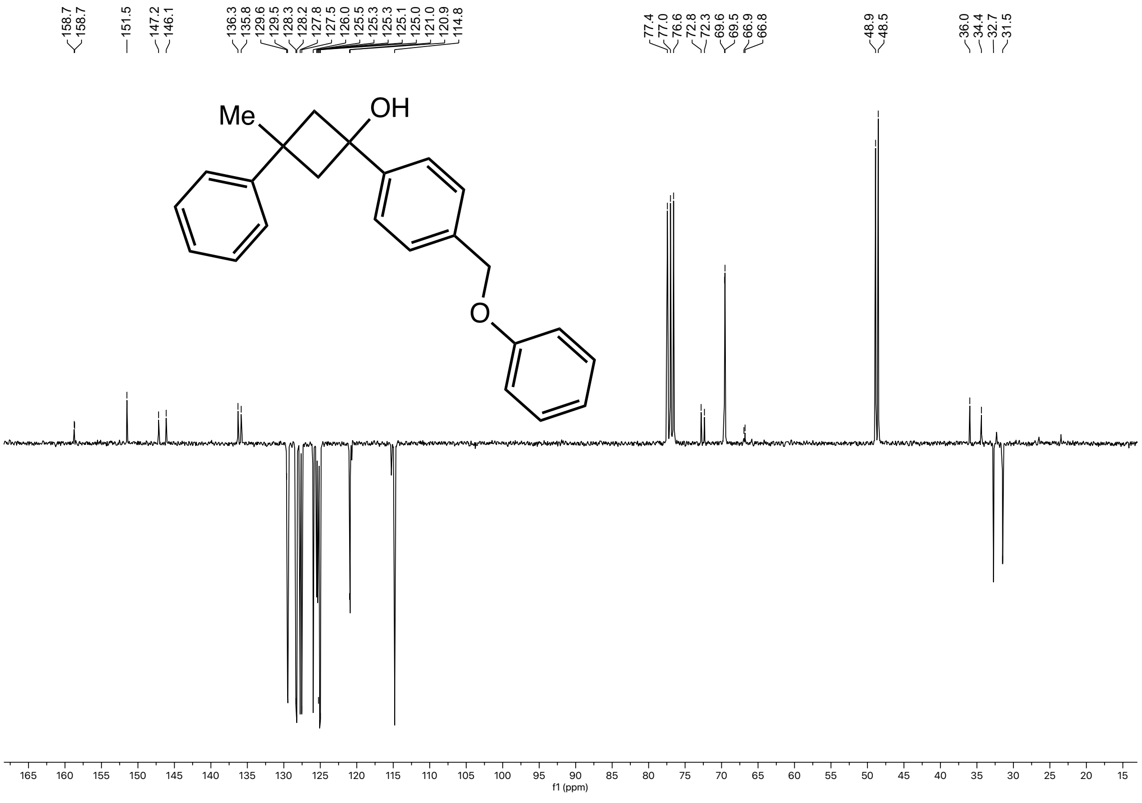

Figure S21. APT $^{13}C\{^{1}H\}$-NMR spectra of 1:1 mixture of diastereoisomers of compound **5** (75.45 MHz, $CDCl_3$)

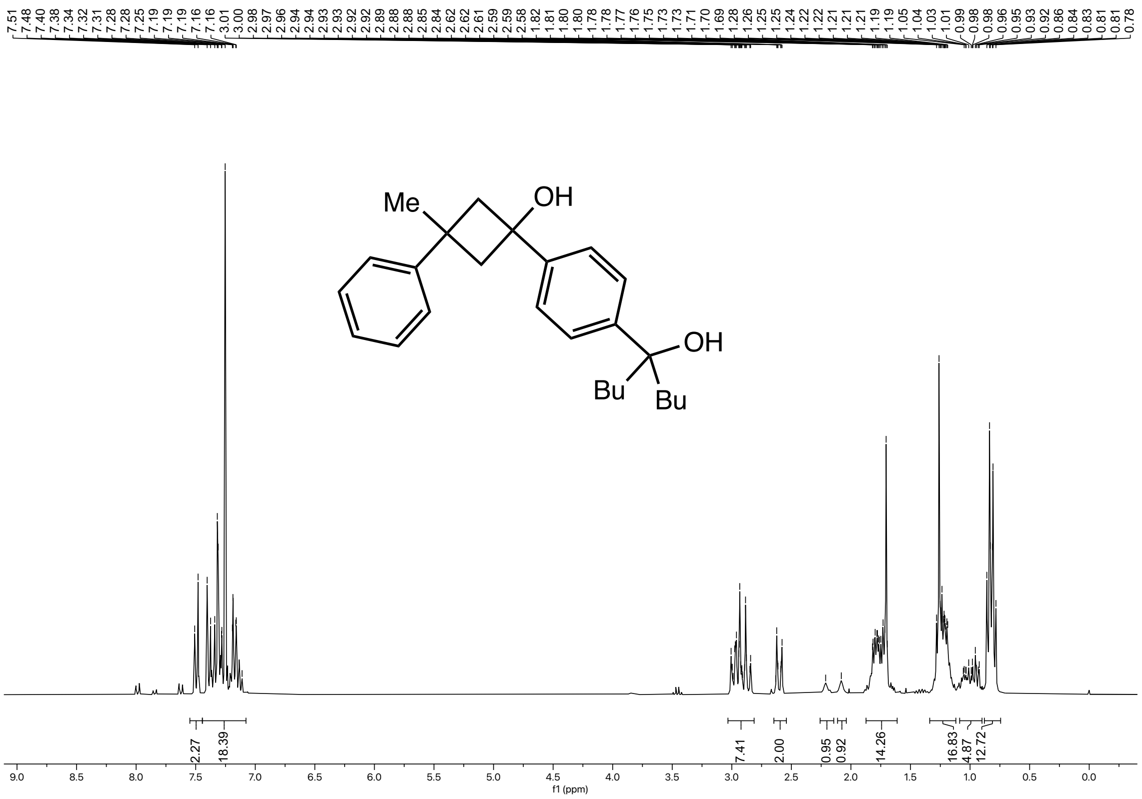

Figure S22. ^{1}H-NMR spectra of 1:0.75 mixture of diastereoisomers of compound **7** (300 MHz, $CDCl_3$)

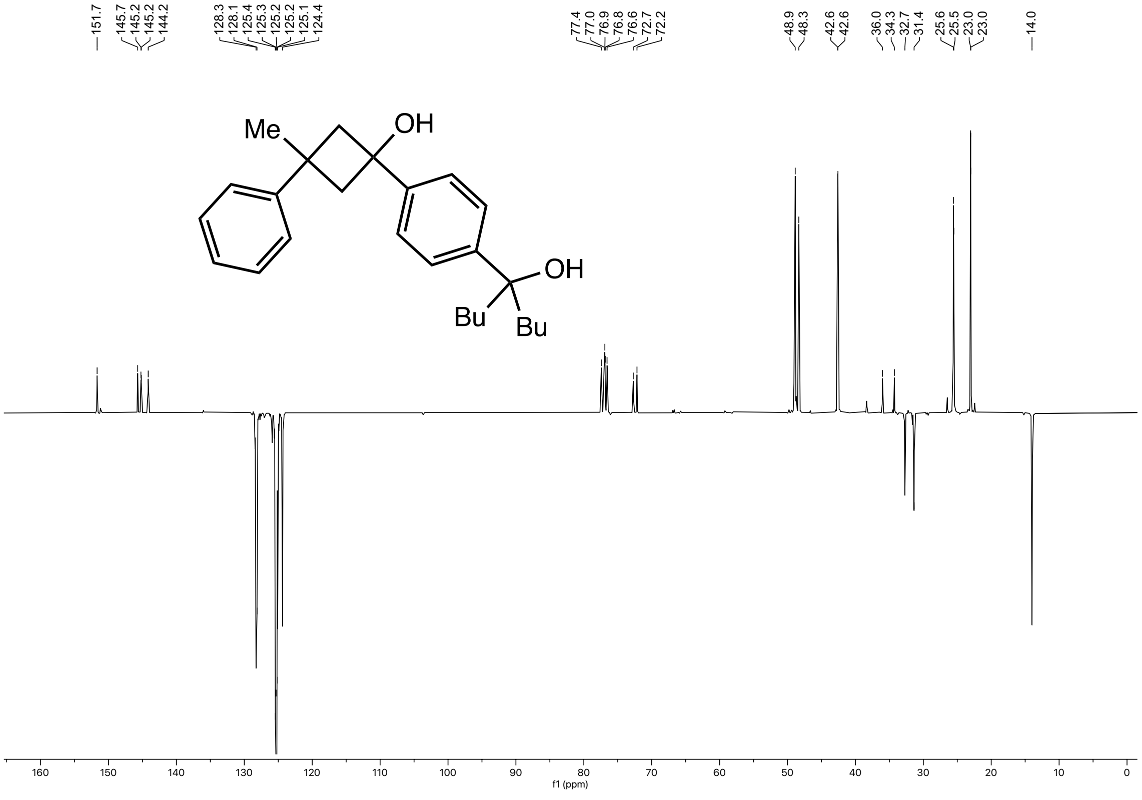

Figure S23. APT $^{13}C\{^{1}H\}$-NMR spectra of 1:0.75 mixture of diastereoisomers of compound **7** (75.45 MHz, $CDCl_3$)

NMR spectra of crude reaction mixture arising from the two-fold C-C bond cleavage procedure and the corresponding purified products

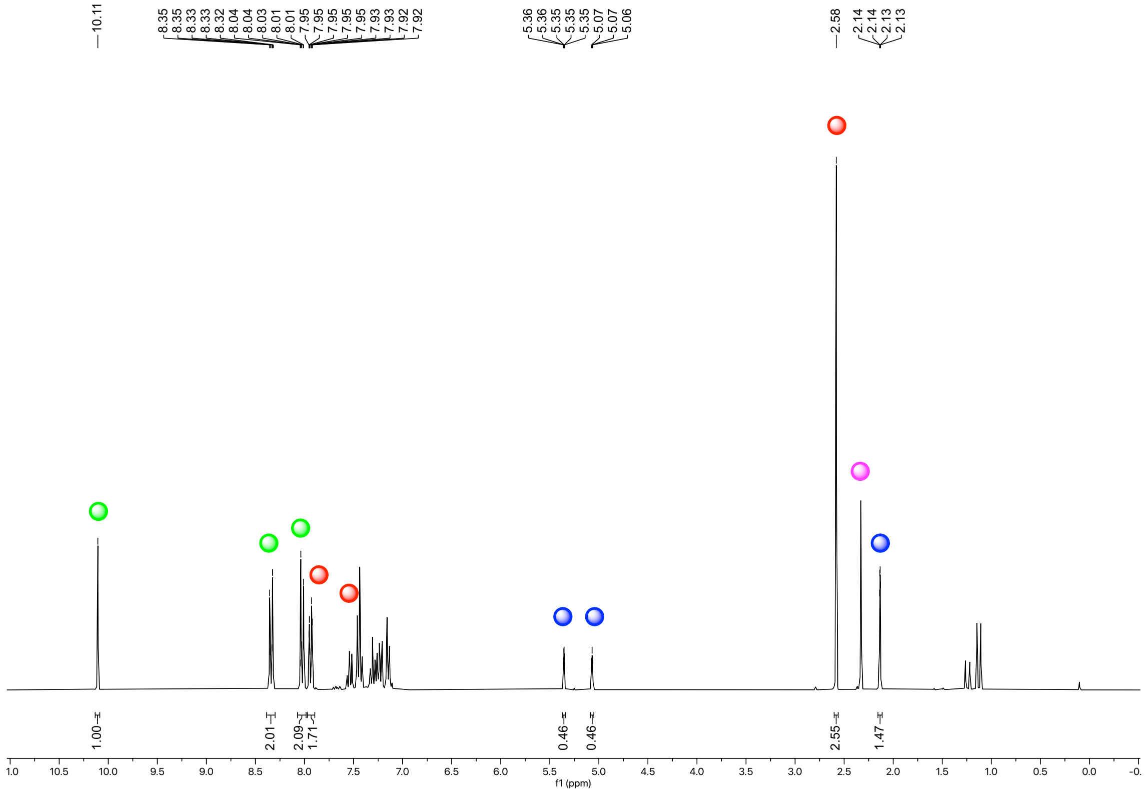

Figure S24a. ^{1}H-NMR spectra of crude reaction mixture arising from the two-fold C-C bond cleavage reaction of compound **1a**, containing signals of acetophenone **2a** (red circle), α-methylstyrene (blue circle), internal standard (4-nitrobenzaldehyde, green circle) and toluene traces (pink circle) (300 MHz, $CDCl_3$).

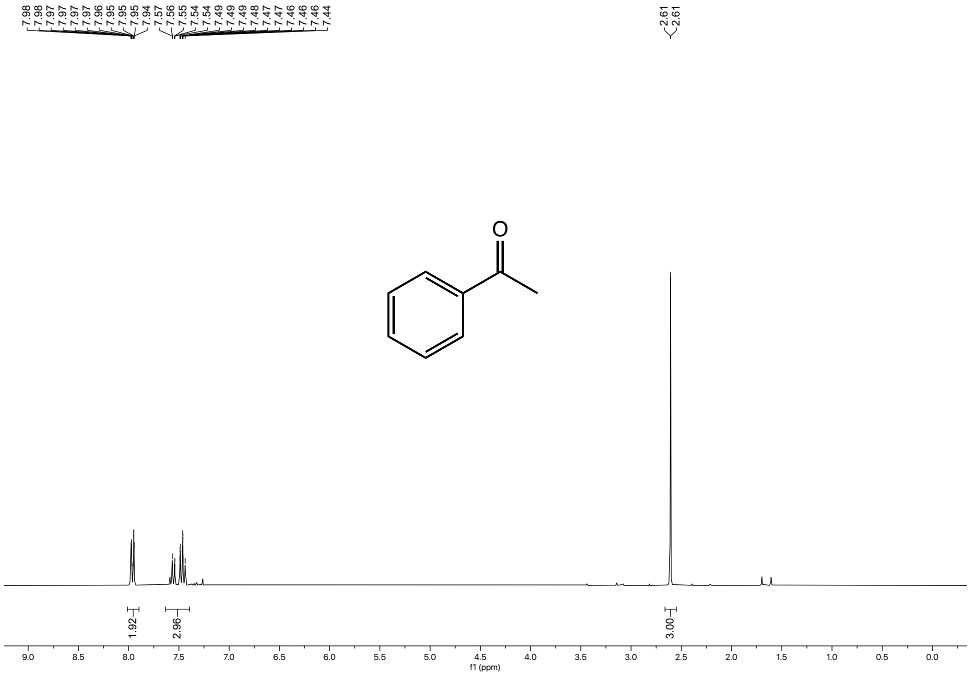

Figure S24b. ^{1}H-NMR spectra of product **2a** (300 MHz, $CDCl_3$).

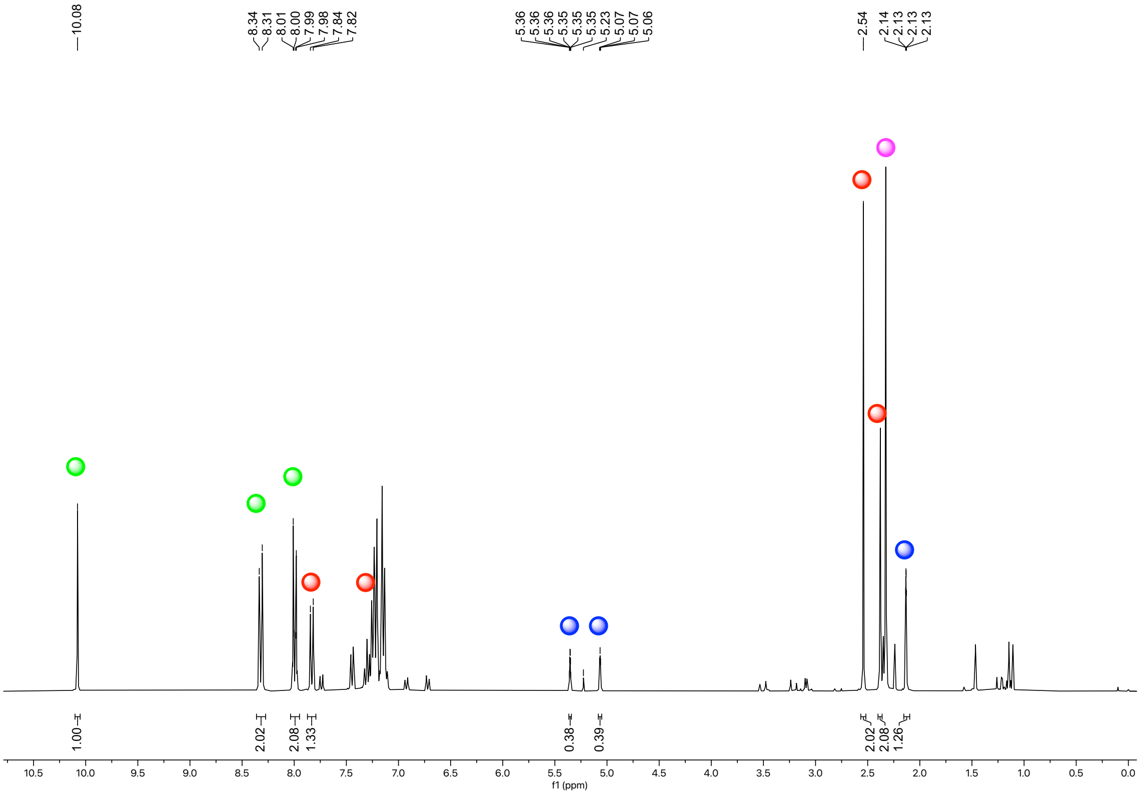

Figure S25a. ^{1}H-NMR spectra of crude reaction mixture arising from the two-fold C-C bond cleavage reaction of compound **1b**, containing signals of acetophenone **2b** (red circle), α-methylstyrene (blue circle), internal standard (4-nitrobenzaldehyde, green circle) and toluene traces (pink circle) (300 MHz, $CDCl_3$).

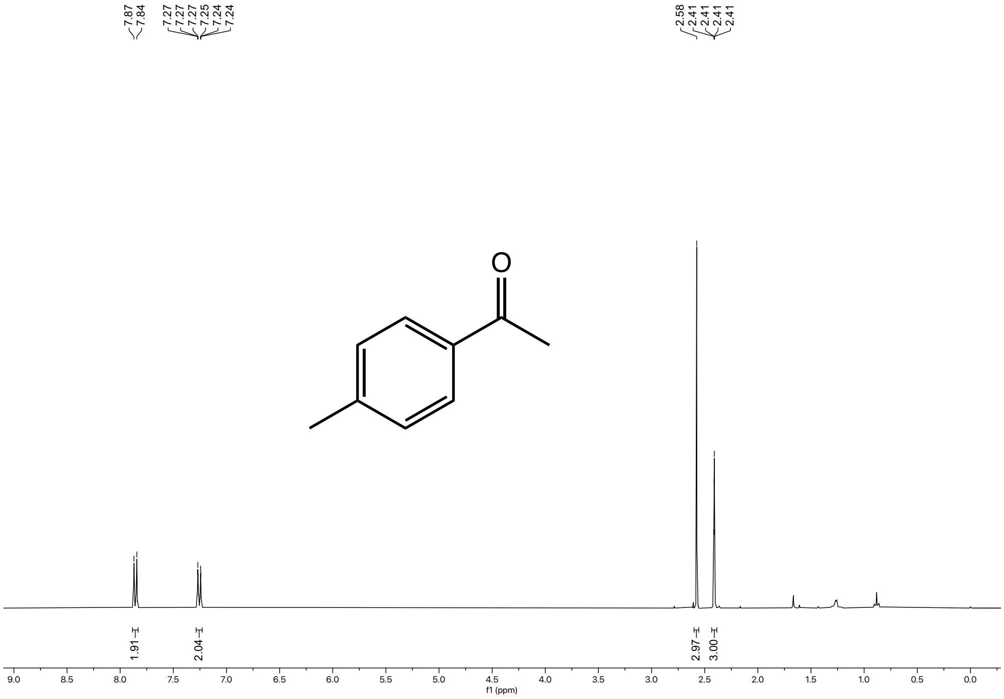

Figure S25b. ^{1}H-NMR spectra of product **2b** (300 MHz, $CDCl_3$).

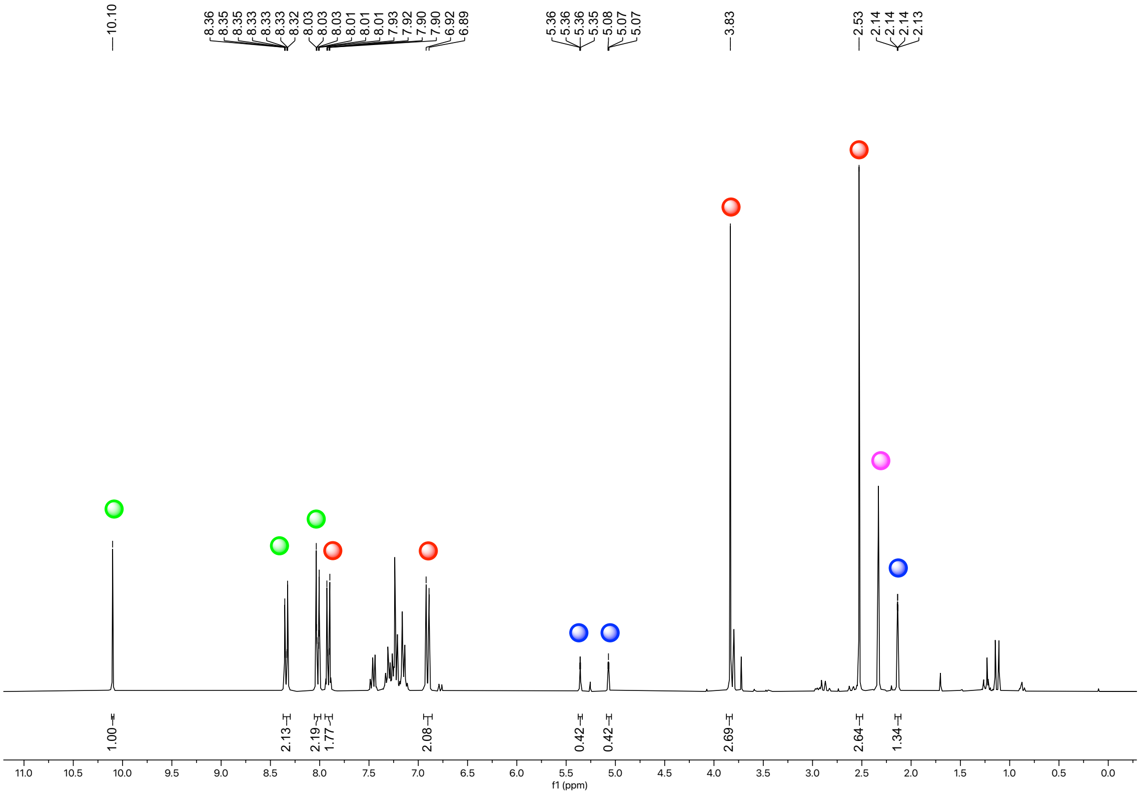

Figure S26a. ^{1}H-NMR spectra of crude reaction mixture arising from the two-fold C-C bond cleavage reaction of compound **1c**, containing signals of acetophenone **2c** (red circle), α-methylstyrene (blue circle), internal standard (4-nitrobenzaldehyde, green circle) and toluene traces (pink circle) (300 MHz, $CDCl_3$).

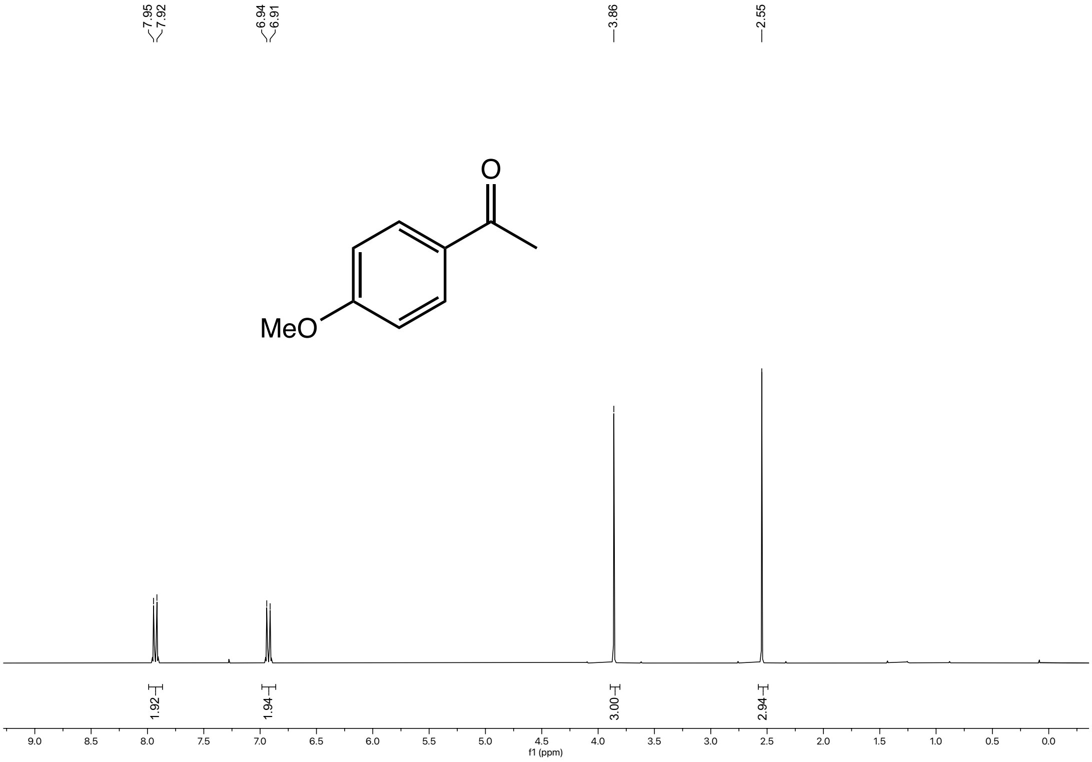

Figure S26b. ^{1}H-NMR spectra of product **2c** (300 MHz, $CDCl_3$).

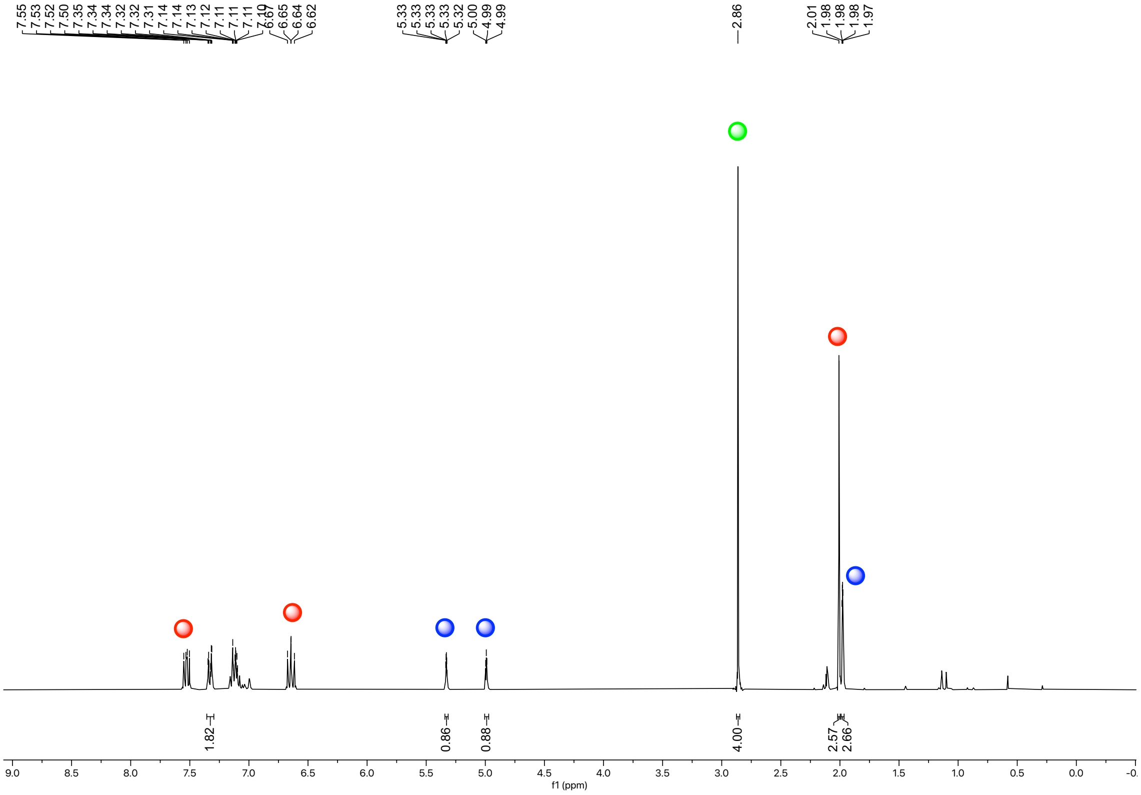

Figure S27a. ^{1}H-NMR spectra of crude reaction mixture arising from the two-fold C-C bond cleavage reaction of compound **1d**, containing signals of acetophenone **2d** (red circle), α-methylstyrene (blue circle) and internal standard (1,2-dibromoethane, green circle) (300 MHz, reaction carried out and checked directly in toluene-d_8).

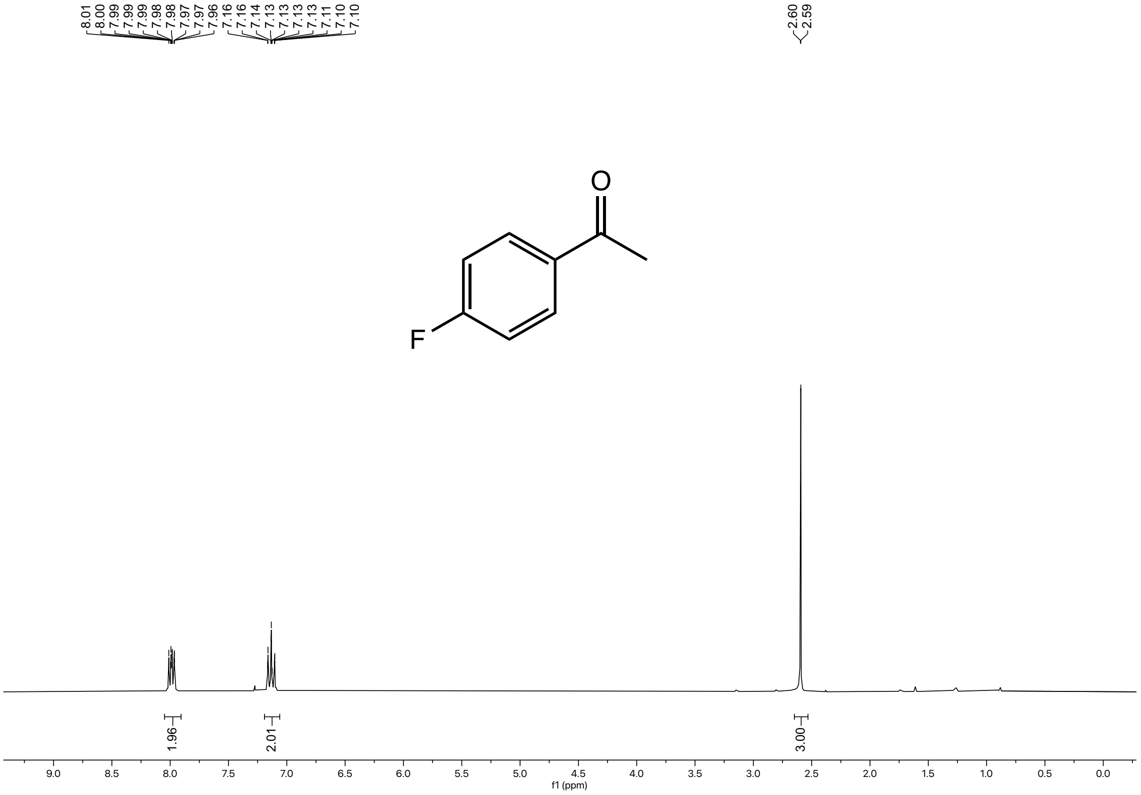

Figure S27b. ^{1}H-NMR spectra of product **2d** (300 MHz, $CDCl_3$).

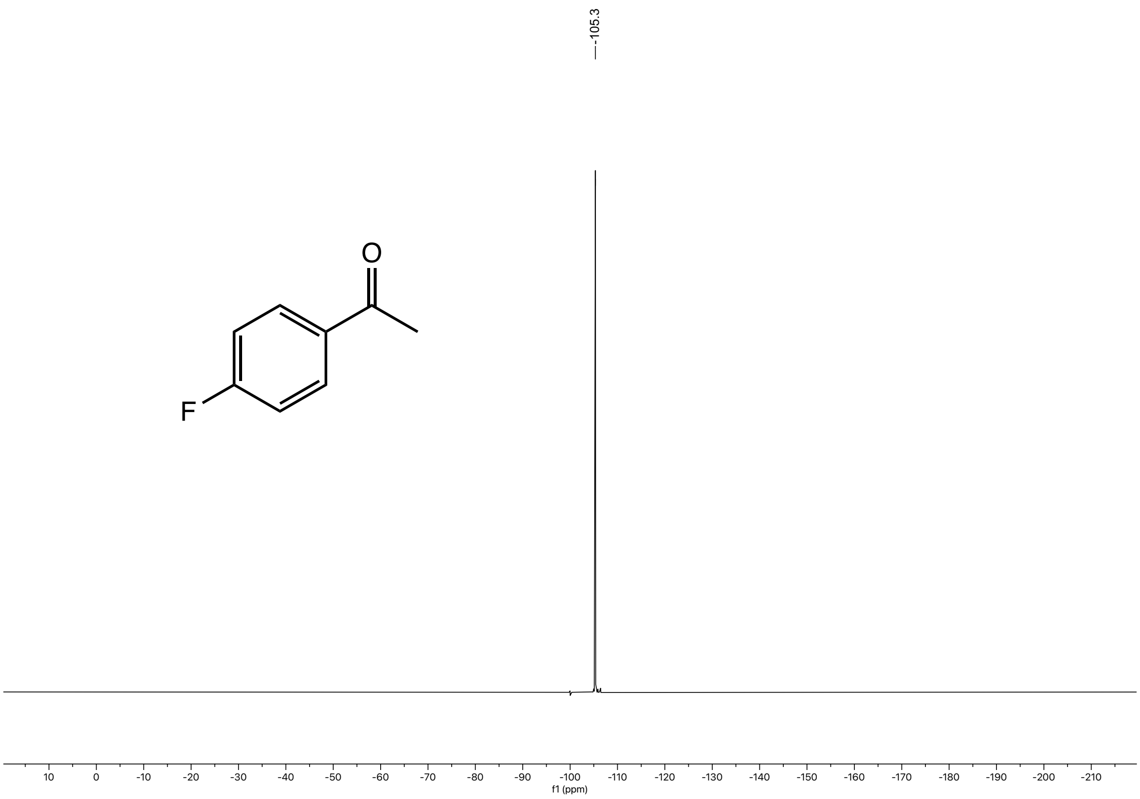

Figure S27c. $^{19}F\{^{1}H\}$-NMR spectra of product **2d** (282.4 MHz, $CDCl_3$).

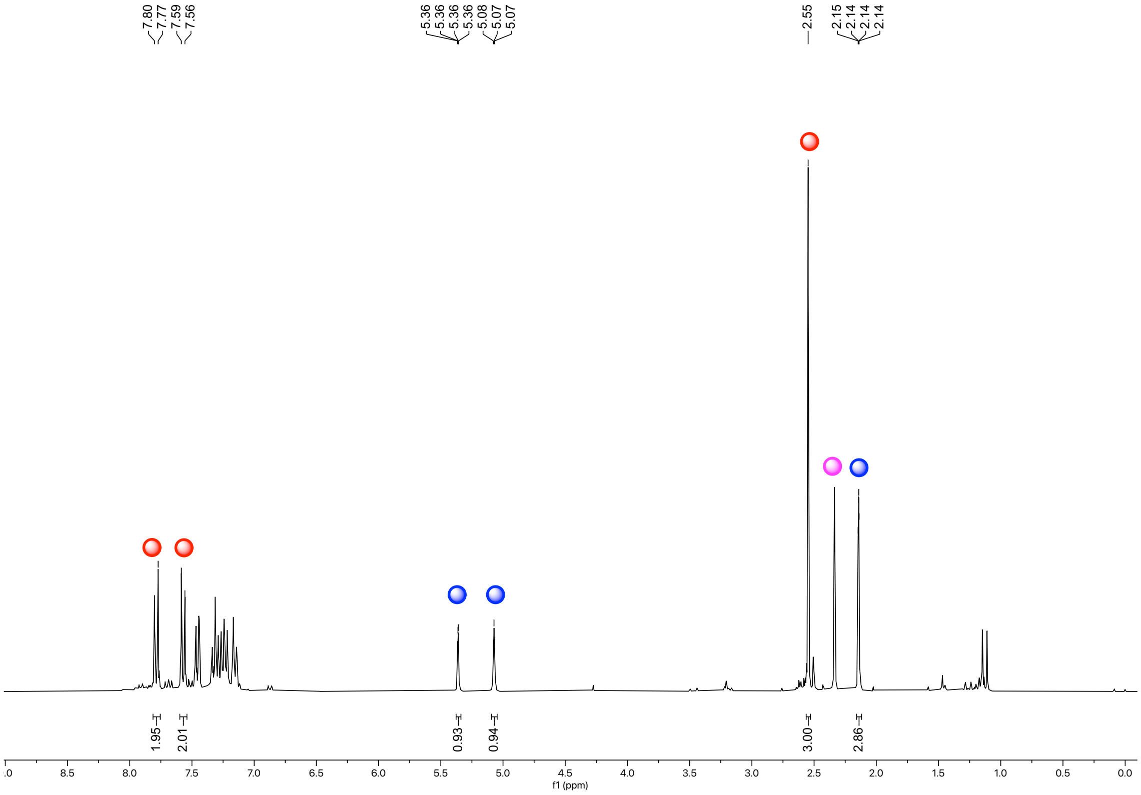

Figure S28a. ^{1}H-NMR spectra of crude reaction mixture arising from the two-fold C-C bond cleavage reaction of compound **1e**, containing signals of acetophenone **2e** (red circle), α-methylstyrene (blue circle) and toluene traces (pink circle) (300 MHz, $CDCl_3$).

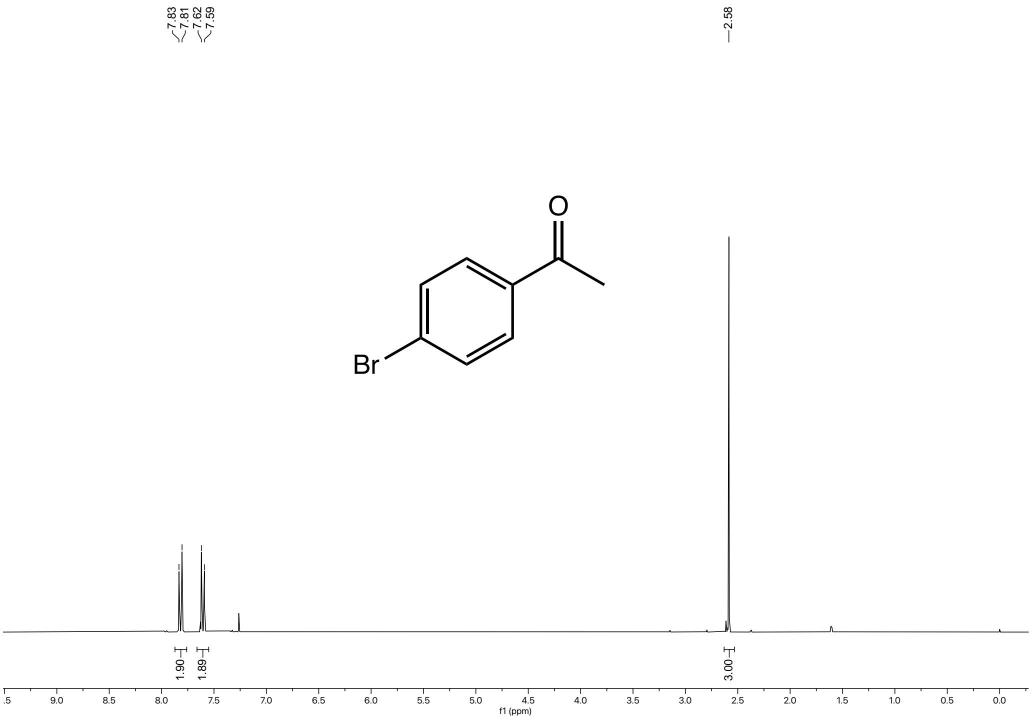

Figure S28b. ^{1}H-NMR spectra of product **2e** (300 MHz, $CDCl_3$).

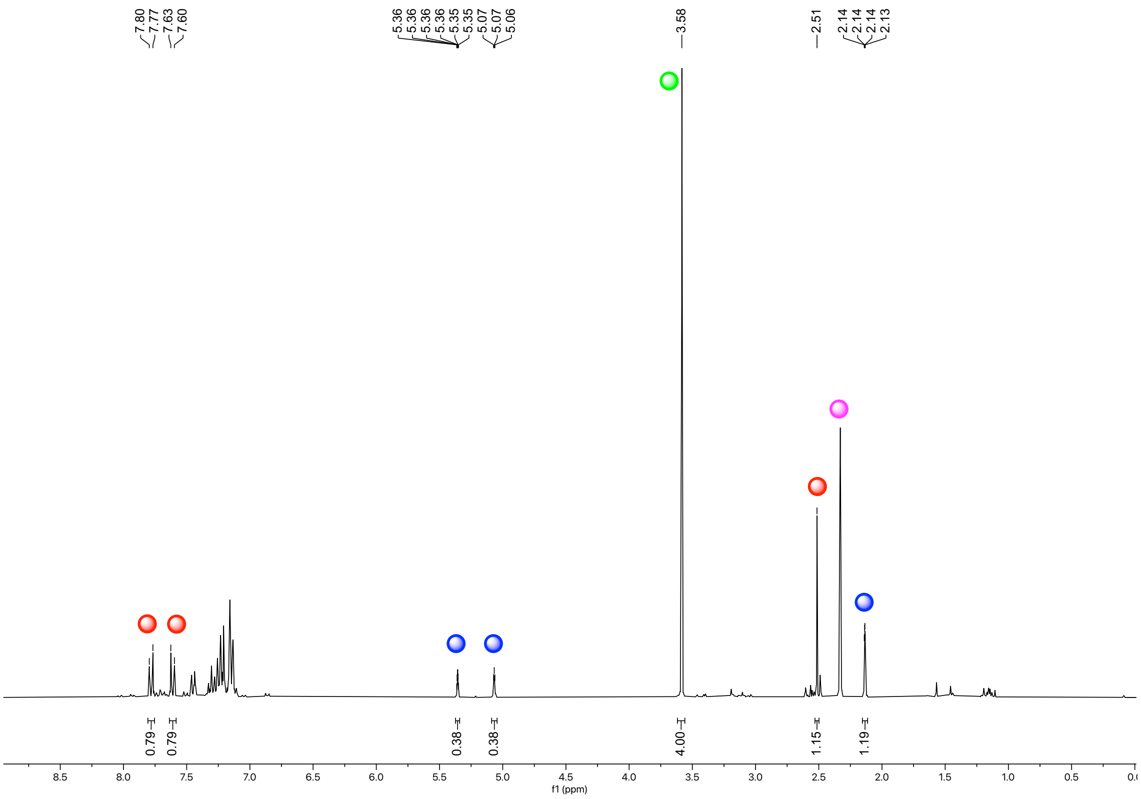

Figure S29a. ^{1}H-NMR spectra of crude reaction mixture arising from the two-fold C-C bond cleavage reaction of compound **1f**, containing signals of acetophenone **2f** (red circle), α-methylstyrene (blue circle), internal standard (1,2-dibromoethane, green circle) and toluene traces (pink circle) (300 MHz, $CDCl_3$).

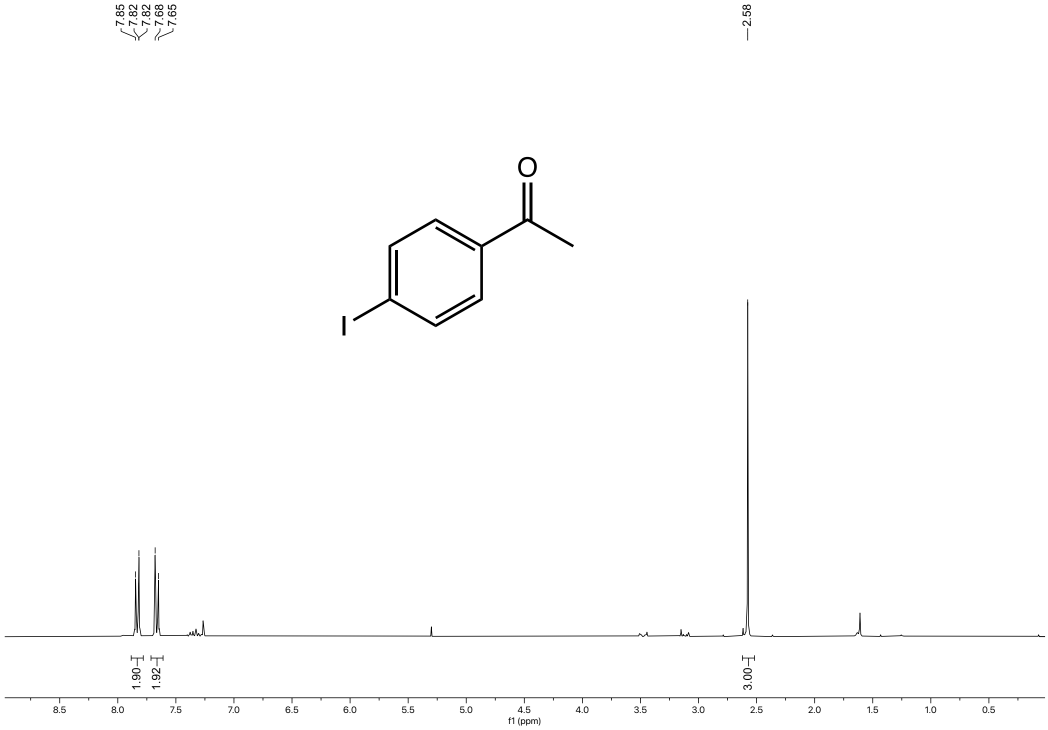

Figure S29b. ^{1}H-NMR spectra of product **2f** (300 MHz, $CDCl_3$).

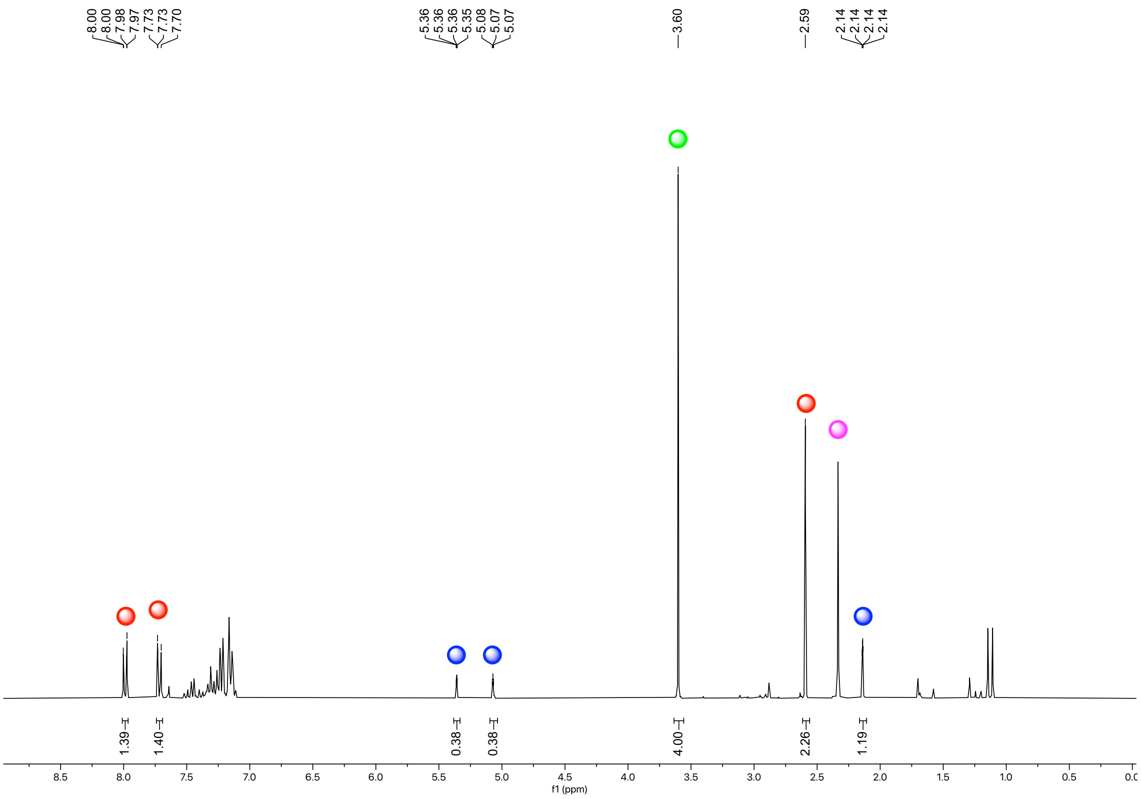

Figure S30a. ^{1}H-NMR spectra of crude reaction mixture arising from the two-fold C-C bond cleavage reaction of compound **1g**, containing signals of the acetophenone **2g** (red circle), α-methylstyrene (blue circle), internal standard (1,2-dibromoethane, green circle) and toluene traces (pink circle) (300 MHz, $CDCl_3$).

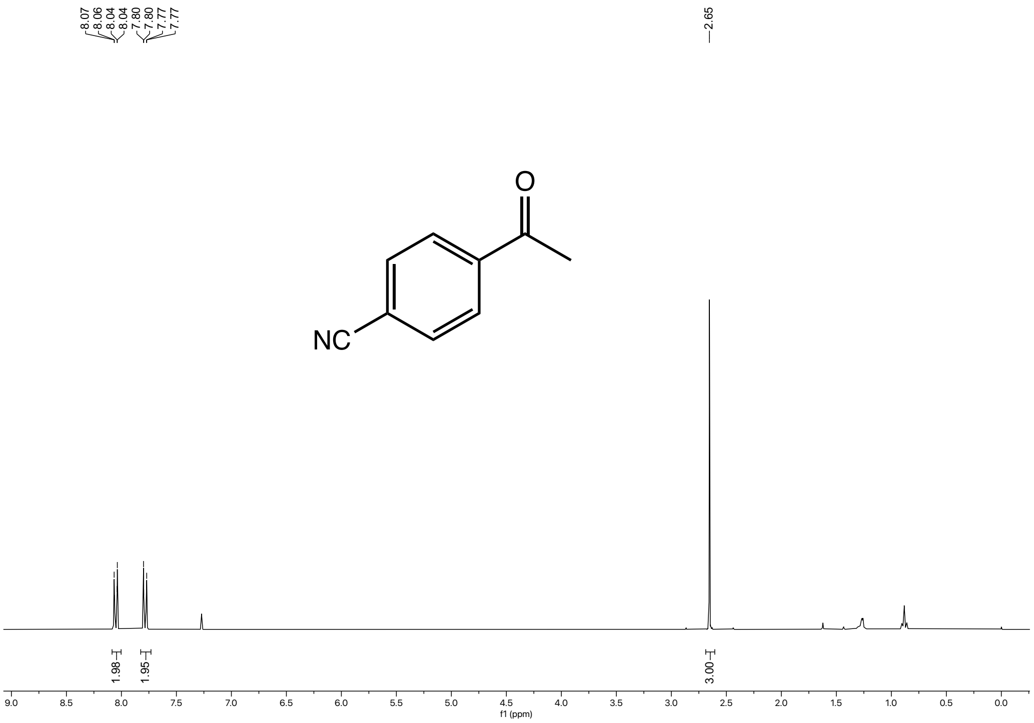

Figure S30b. ^{1}H-NMR spectra of product **2g** (300 MHz, $CDCl_3$).

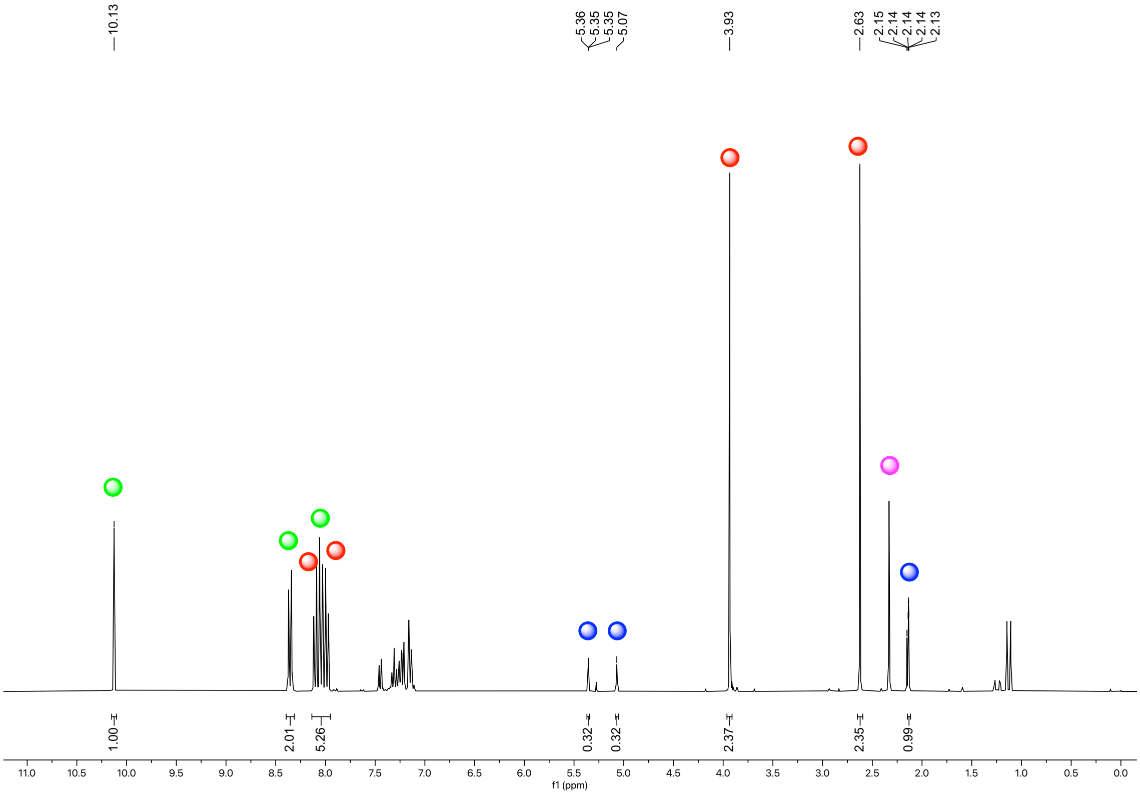

Figure S31a. ^{1}H-NMR spectra of crude reaction mixture arising from the two-fold C-C bond cleavage reaction of compound **1h**, containing signals of acetophenone **2h** (red circle), α-methylstyrene (blue circle), internal standard (4-nitrobenzaldehyde, green circle) and toluene traces (pink circle) (300 MHz, $CDCl_3$).

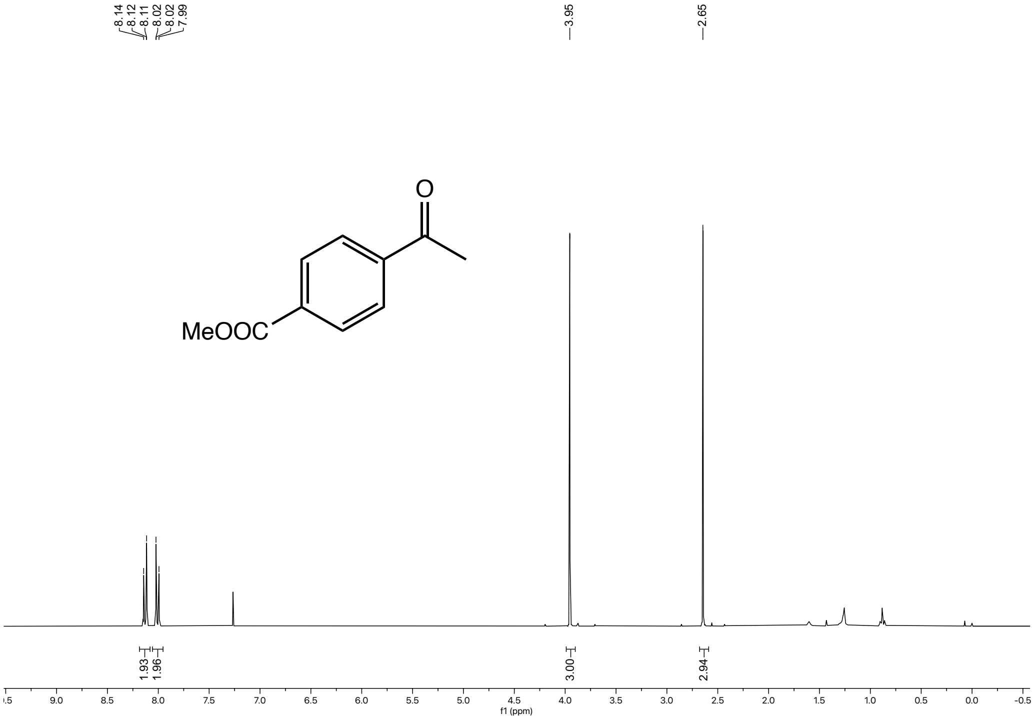

Figure S31b. ^{1}H-NMR spectra of product **2h** (300 MHz, $CDCl_3$).

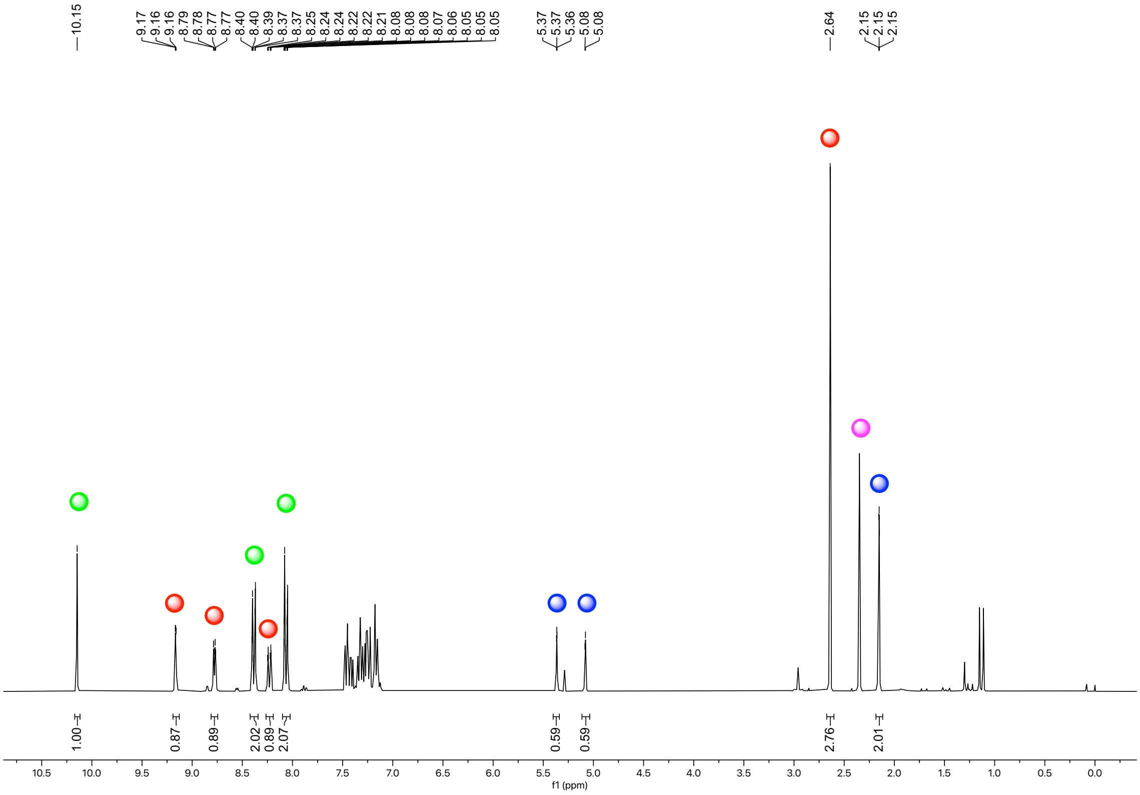

Figure S32a. ^{1}H-NMR spectra of crude reaction mixture arising from the two-fold C-C bond cleavage reaction of compound **1i**, containing signals of product **2i** (red circle), α-methylstyrene (blue circle), internal standard (4-nitrobenzaldehyde, green circle) and toluene traces (pink circle) (300 MHz, $CDCl_3$).

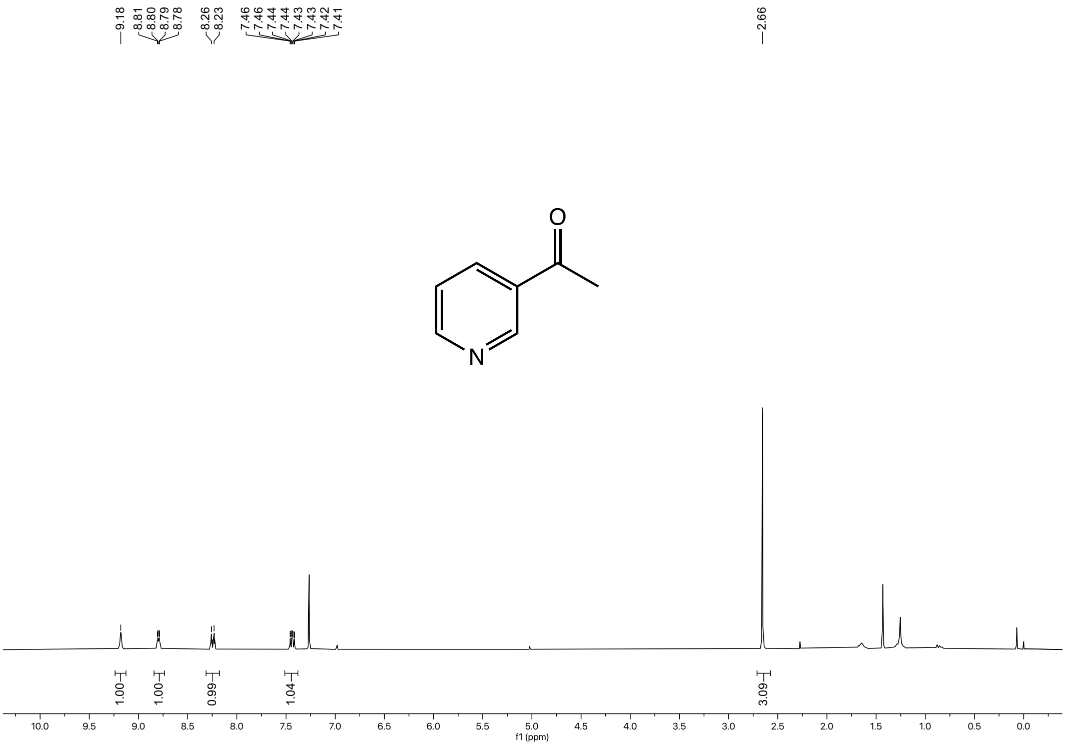

Figure S32b. ^{1}H-NMR spectra of product **2i** (300 MHz, $CDCl_3$).

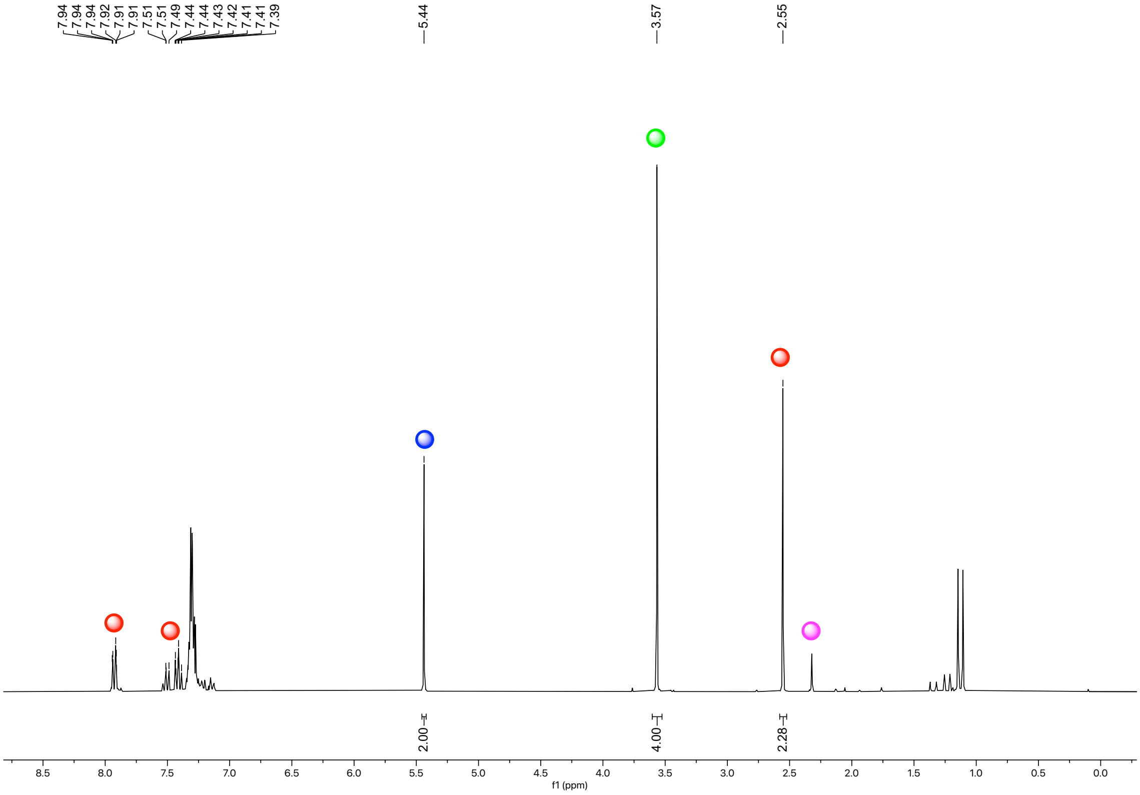

Figure S33a. ^{1}H-NMR spectra of crude reaction mixture arising from the two-fold C-C bond cleavage reaction of compound **1n** with 10 mol% of $Pd(OAc)_2$, containing signals of acetophenone **2a** (red circle), alkene **3b** (blue circle), internal standard (1,2-dibromoethane, green circle) and toluene traces (pink circle) (300 MHz, $CDCl_3$).

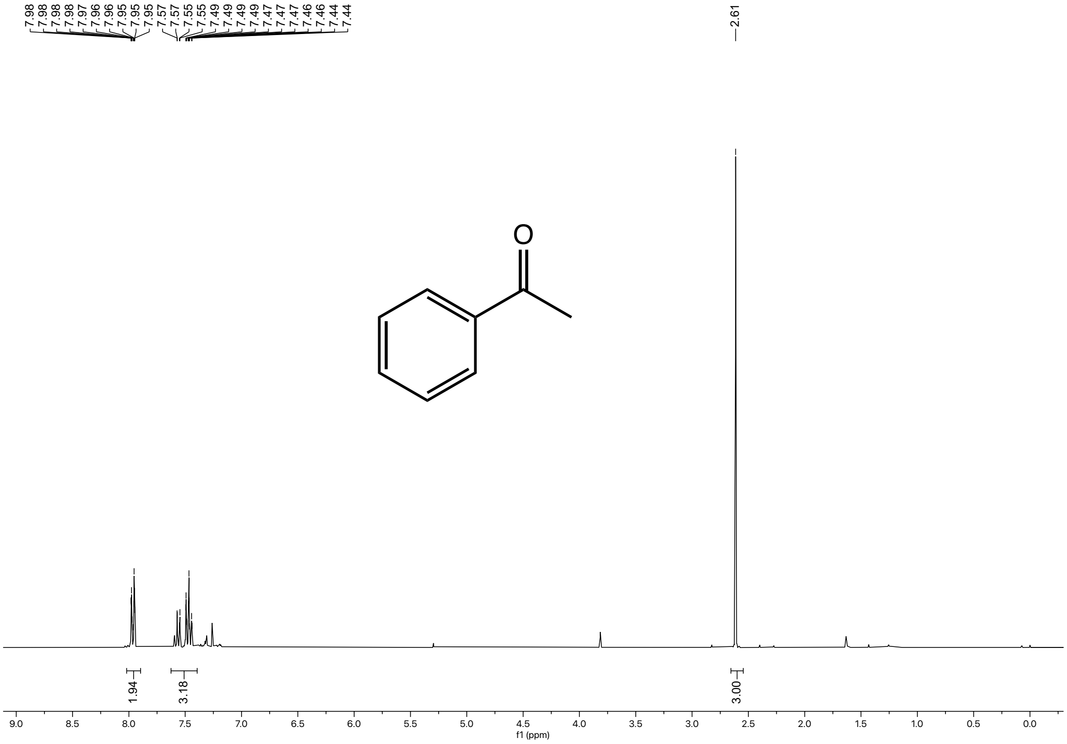

Figure S33b. ^{1}H-NMR spectra of product **2a** (300 MHz, $CDCl_3$).

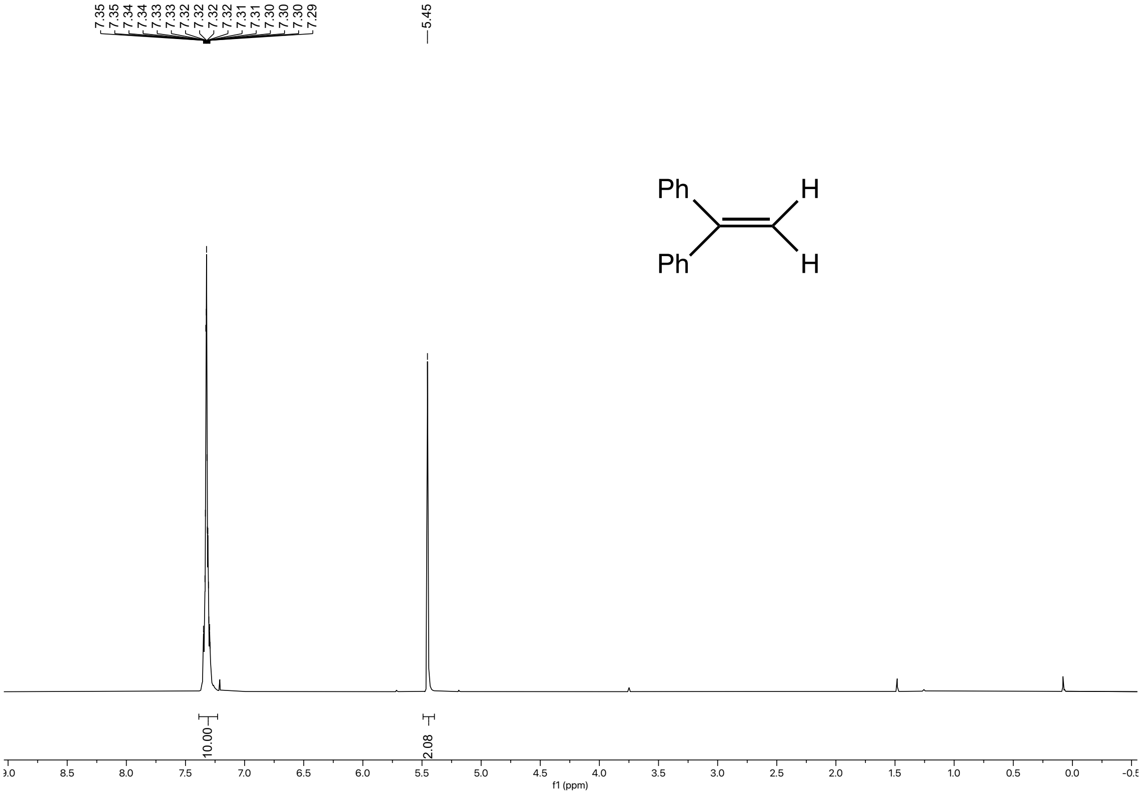

Figure S33c. ^{1}H-NMR spectra of alkene **3b** (300 MHz, $CDCl_3$).

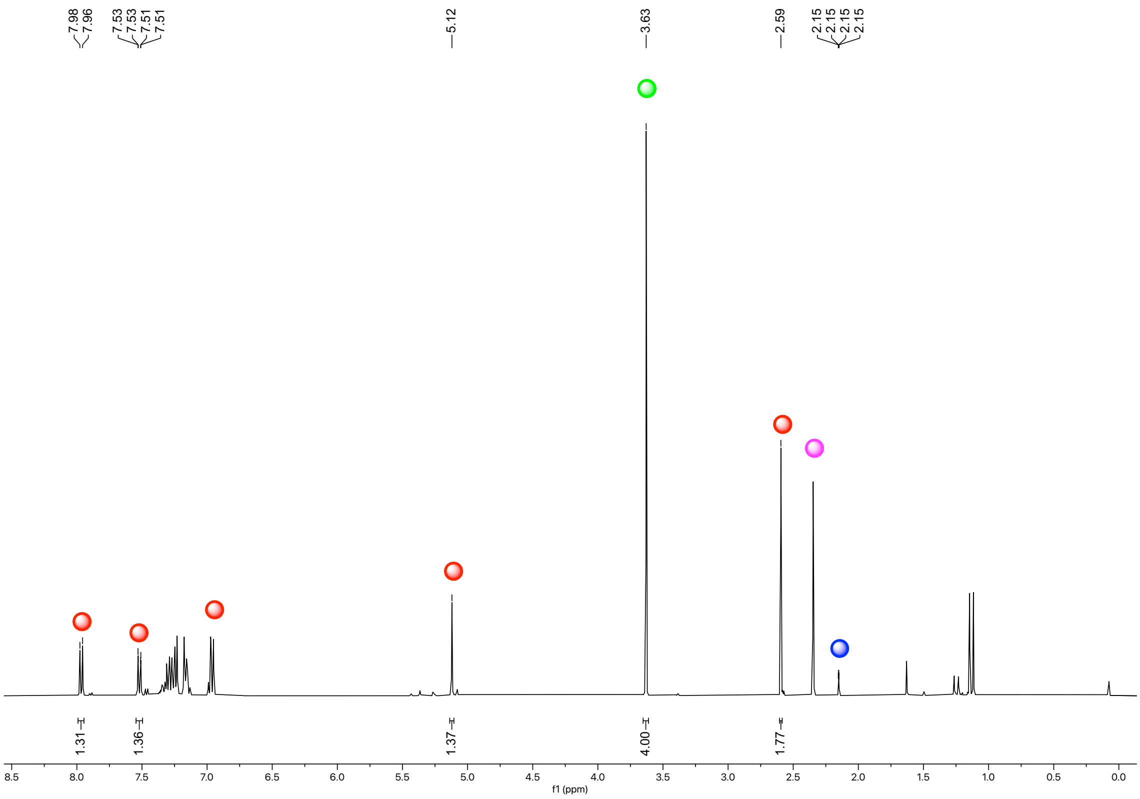

Figure S34a. ^{1}H-NMR spectra of crude reaction mixture arising from the two-fold C-C bond cleavage reaction of compound **5** with 8 mol% of $Pd(OAc)_2$, containing signals of acetophenone **6** (red circle), α-methylstyrene (blue circle), internal standard (1,2-dibromoethane, green circle) and toluene traces (pink circle) (400 MHz, $CDCl_3$).

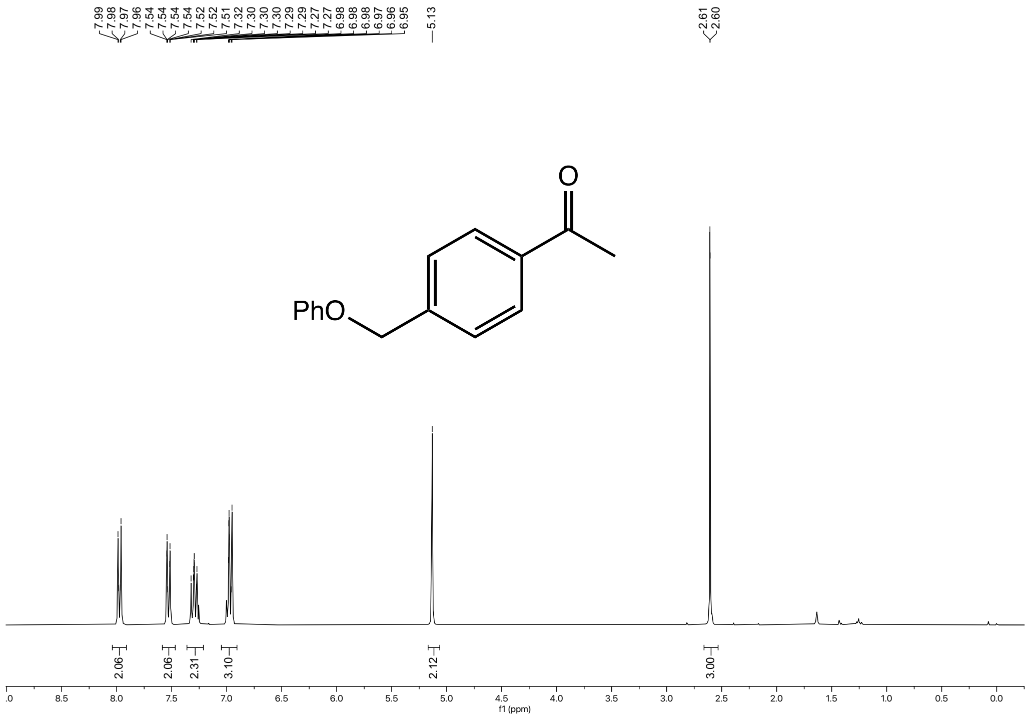

Figure S34b. ^{1}H-NMR spectra of product **6** (300 MHz, $CDCl_3$).

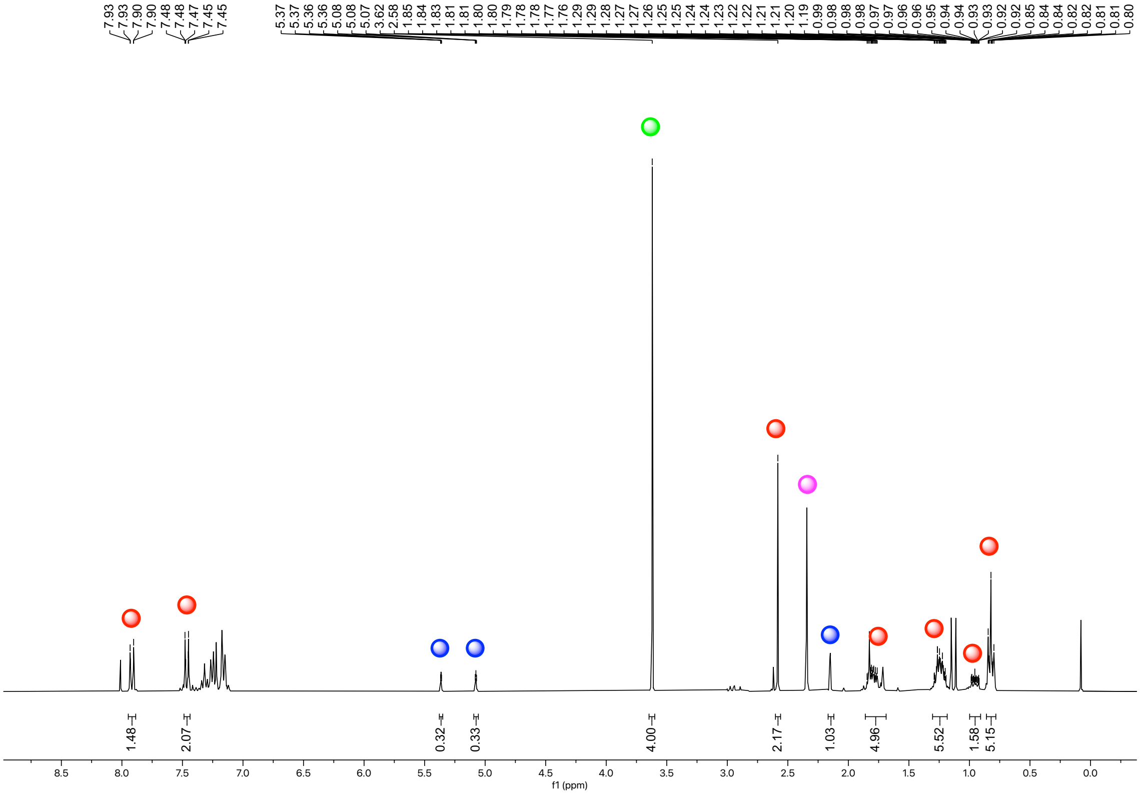

Figure S35a. ^{1}H-NMR spectra of crude reaction mixture arising from the two-fold C-C bond cleavage reaction of compound **7** with 5 mol% of $Pd(OAc)_2$, containing signals of acetophenone **8** (red circle), α-methylstyrene (blue circle), internal standard (1,2-dibromoethane, green circle) and toluene traces (pink circle) (300 MHz, $CDCl_3$).

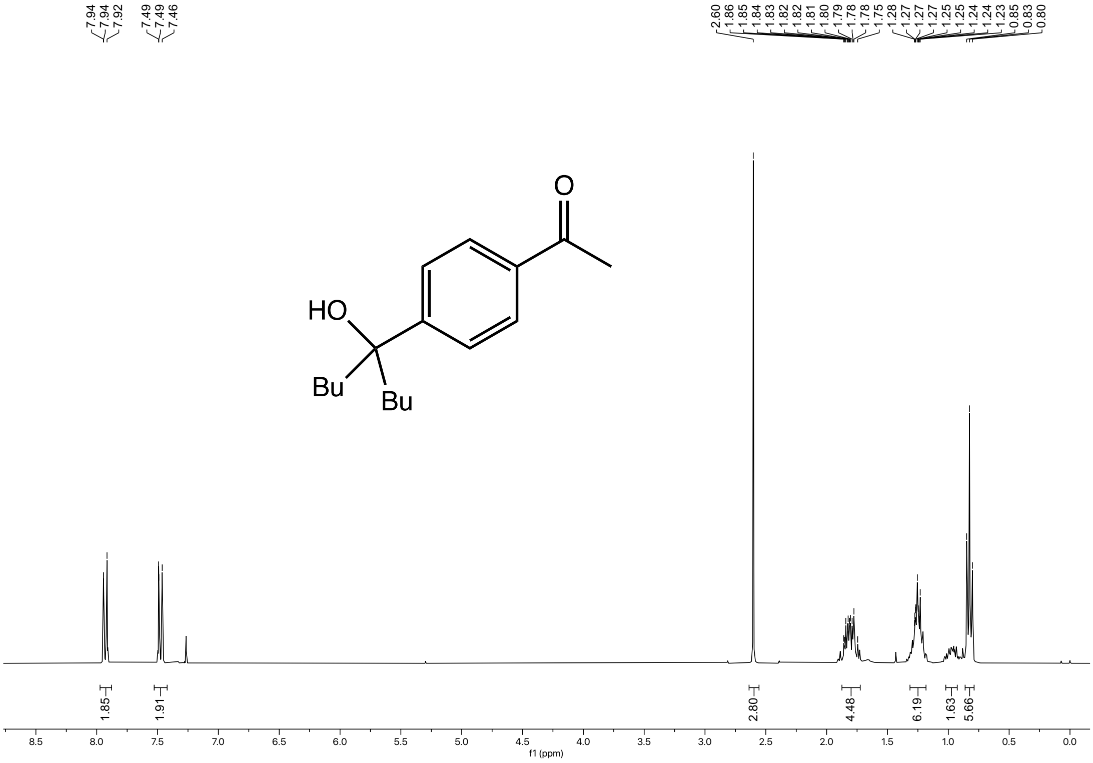

Figure S35b. ^{1}H-NMR spectra of product **8** (300 MHz, $CDCl_3$).
